# Memory of cell shape biases stochastic fate decision-making despite mitotic rounding

**DOI:** 10.1038/ncomms11963

**Published:** 2016-06-28

**Authors:** Takashi Akanuma, Cong Chen, Tetsuo Sato, Roeland M. H. Merks, Thomas N. Sato

**Affiliations:** 1 The Thomas N. Sato BioMEC-X Laboratories, Advanced Telecommunications Research Institute International (ATR), Hikaridai 2-2-2, Kyoto 619-0288, Japan; 2 Centrum Wiskunde & Informatica, Life Sciences Group, Science Park 123, 1098 XG, Amsterdam, The Netherlands; 3 Section Computational Science, Informatics Institute, University of Amsterdam, Science Park 904, 1098 XH Amsterdam, The Netherlands; 4 Graduate School of Information Science, Nara Institute of Science and Technology, Takayama 8916-5, Ikoma-shi, Nara 630-0192, Japan; 5 Mathematical Institute, Research Programme Analysis and Dynamical Systems, Leiden University, P.O. Box 9512, 2300 RA Leiden, The Netherlands; 6 ERATO Sato Live Bio-Forecasting Project, Japan Science and Technology Agency (JST), Hikaridai 2-2-2, Kyoto 619-0288, Japan; 7 Department of Biomedical Engineering, Cornell University, 101 Weill Hall, Ithaca, New York 14853, USA; 8 Centenary Institute, Sydney, Locked Bag 6, Newtown, New South Wales 2042, Australia

## Abstract

Cell shape influences function, and the current model suggests that such shape effect is transient. However, cells dynamically change their shapes, thus, the critical question is whether shape information remains influential on future cell function even after the original shape is lost. We address this question by integrating experimental and computational approaches. Quantitative live imaging of asymmetric cell-fate decision-making and their live shape manipulation demonstrates that cellular eccentricity of progenitor cell indeed biases stochastic fate decisions of daughter cells despite mitotic rounding. Modelling and simulation indicates that polarized localization of Delta protein instructs by the progenitor eccentricity is an origin of the bias. Simulation with varying parameters predicts that diffusion rate and abundance of Delta molecules quantitatively influence the bias. These predictions are experimentally validated by physical and genetic methods, showing that cells exploit a mechanism reported herein to influence their future fates based on their past shape despite dynamic shape changes.

## Abstract (web summary)

Cell shape influences function but during mitotic cell rounding the original shape is lost. Here the authors show that the cellular eccentricity of progenitor cell biases stochastic fate-decisions using a combination of quantitative live imaging, genetic manipulations and computational simulations.

## Graphical abstract

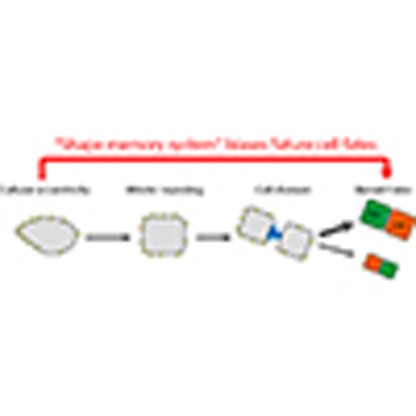

The interdependence of cell shape and cell function is a central and long-lasting question in biology. The importance of cell shape in cellular function has been recognized for centuries and has fascinated a number of scientists and thus has precipitated many studies. Cells of distinct functions exhibit unique shapes. Both intrinsic genetic programmes and extracellular microenvironment of the cells regulate intracellular signals, which eventually modulate cell shape. Cells of distinct lineages, cells of different organs and different cell types in an organ can be identified by their morphological differences. Furthermore, such relation is also exploited in medical diagnosis. Malignant cells and/or dysfunctional cells could be often identified by their peculiar shapes.

In addition to such functional and/or phenotypic influences of the cells on their shapes (that is, function→shape relation), shapes also influence intracellular signals and functions (that is, shape→function relation). The classical example is Hertwig's rule (a.k.a. long-axis rule). This is an empirical rule proposed by Hertwig based on his studies of echinoderm and frog eggs. This rule posits that cells divide at their cytoplasmic centre perpendicular to their longest axis^1^. While the original Hertwig's rule is applicable to symmetrical cells such as echinoderm and frog eggs, its more general applicability to the cells of diverse shapes remained unknown until recently. Furthermore, the original rule lacked quantitative descriptions of cell shapes or axis. These problems were recently addressed by Minc *et al*., with a combination of sophisticated shape manipulation experiments and a theoretical approach, resulting in the generalization and quantitative description of the rule^2^,^3^. An early experimental study altered cell shapes by varying substrate adhesiveness and examined DNA synthesis and cell proliferation, finding that cell shape controls DNA synthesis and proliferation^4^. Another study uses a mathematical model to examine the effect of cell size and shape on the activation of G-protein-mediated intracellular signal. The model suggested that as the cell size increases, the signal transduction is attenuated. In contrast, the model indicated that as the cell becomes spreads and flattened, the signal is enhanced. Furthermore, the model predicted that the signals also become enhanced in the leading edge of polarized cells and in cellular protrusions^5^. These predictions were also experimentally validated using cultured cells^5^. The critical importance of cell shape in cellular morphogenesis is also implicated^6^,^7^. The study used micropatterning of an adhesion promoter and substrate fabrication methods to influence by design cellular geometry and showed that cell shape regulates cell polarity and ciliogenesis^6^. More recent study, using analytical approaches and numerical simulation, studied how cell shape elongation influences plasma membrane signalling^8^. The mathematical analyses revealed that activated signal-transducing receptors accumulate at the region of higher curvature of plasma membrane, with increasing cellular eccentricity^8^. Numerical simulations showed that the accumulation of the activated receptors at the region of higher curvature amplifies downstream signalling activities^8^. These theoretical predictions were experimentally validated by altering cellular eccentricity, showing that plasma membrane is a locus of shape information storage and retrieval^8^.

While these previous studies clearly show that cell shape influences cellular function, the current models suggest that the information storage in shape is transient^8^,^9^. Cells dynamically change their shapes, especially *in vivo* through mitotic rounding, division, differentiation, migration, and cell–cell and cell–extracellular matrix interactions. Hence, it is critical to determine whether shape information could be retained as a ‘memory' for an extended period of time for later retrieval and used to instruct cell function and/or fate, even after the cell no longer retains the original shape through dynamic shape changes.

Here we address this question by integrating computational and experimental approaches. As a model system, we study asymmetric fate decision-making^10^. In asymmetric fate decision-making, a single progenitor cell produces two daughter cells of distinct functions and phenotypes through mitotic rounding followed by cell division. Through the mitotic rounding, the original shape of the progenitor cell is lost. Hence, the effects of the progenitor cell shape no longer persist in the daughter cells according the current transient-effect model^8^,^9^. The fates or the phenotypes of the daughter cells are indeed unknown before their generation after the division^10^. Therefore, the question we raised is that whether we can predict the outcome of the daughter cell fates based on the progenitor cell shape before mitotic rounding and cell division. Among many asymmetric fate decision-making models, we choose asymmetric fate decision-making of V2 neural progenitor cells (V2 cells) in developing zebrafish nervous system^11^. In this model system, each V2 cell undergoes mitotic rounding and division to produce two phenotypically and functionally distinct daughter cells, V2a and V2b, and the V2a/V2b fate decisions appear to be stochastic^11^. Furthermore, zebrafish embryos are easily accessible for live imaging and femtosecond laser mediated shape manipulation. Therefore, this system provides an ideal *in vivo* system to study the effects of original cell shape on fate decisions of the cell following dynamic shape changes such as mitotic rounding.

Quantitative live imaging of individual V2 progenitor cells and their daughter cells shows that bias in the daughter cell fates can indeed be predicted by the orientation and degree of the elongation of each V2 progenitor cell. Femtosecond laser-mediated shape manipulation experiment demonstrates that cellular eccentricity of V2 progenitor cells is causal in imposing bias on the daughter cell fates. Modelling and computational simulation studies suggest that polarized localization of Delta protein towards the edge of the cellular elongation of V2 progenitor cell is an origin of the bias despite the diffusion of Delta protein during mitotic rounding. Computational simulations predict that shape instructs the polarized localization of Delta protein and its abundance and the rate of its diffusion critically and quantitatively influence the bias. These predictions are experimentally validated by femtosecond laser-mediated shape manipulation and loss-of- and gain-of-function approaches, illustrating a mechanism that cells exploit to influence their future fates based on their past shape despite dynamic shape changes such as mitotic rounding.

## Results

### Cellular eccentricity of V2 cells biases their fates

**Figure 1:**
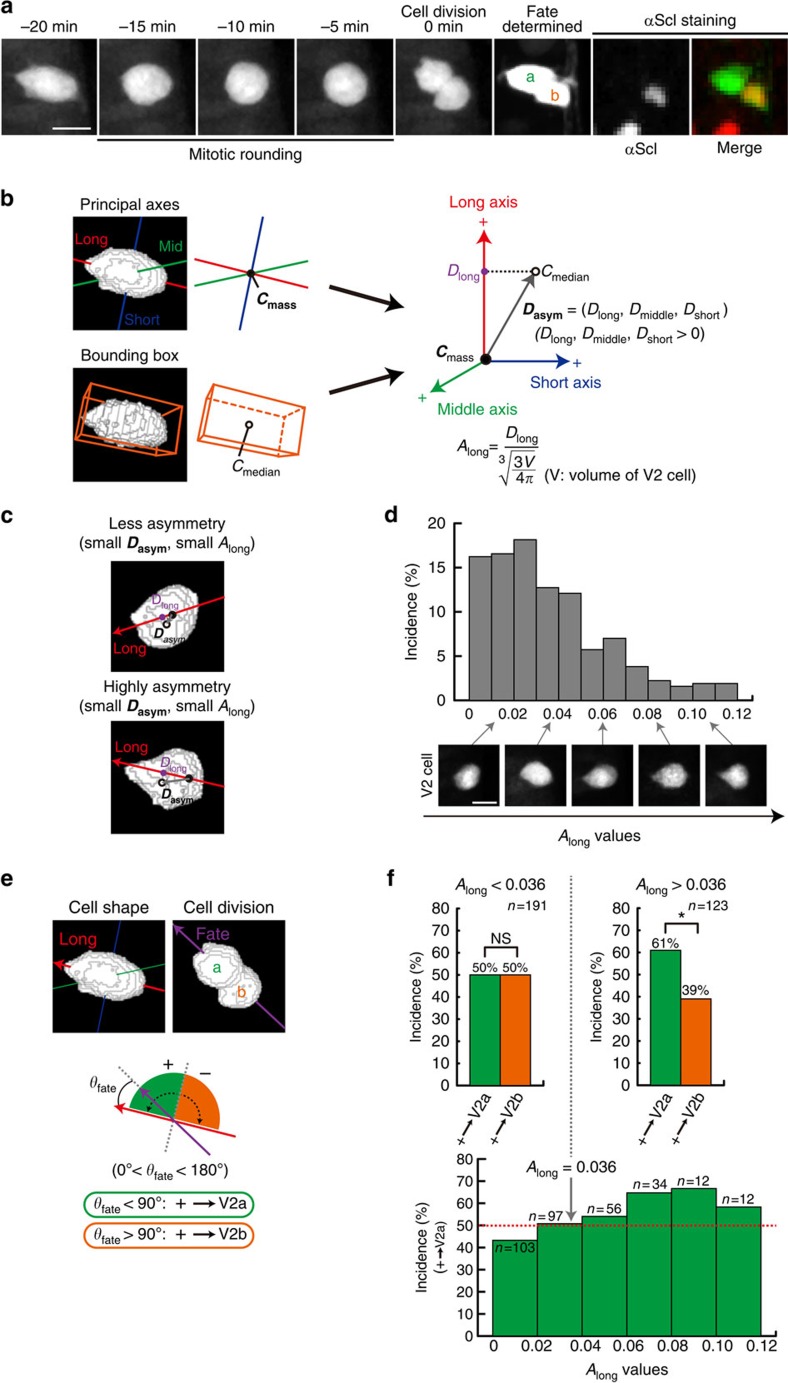
Quantitative definition of cellular eccentricity of V2 cells and its relation to their fates. (**a**) Dynamics of shape changes of V2 cell. (**b**) Quantitative representation of the direction and degree of the asymmetric elongation of V2 cell shape. (**c**) Typical examples of less (top) and highly (bottom) asymmetric cell shapes in 3D. (**d**) The distribution of various *A*_long_ values of all V2 cells (*n*=314) analysed in this study. See also Supplementary Fig. 1 and Supplementary Movies 1 and 2. (**e**) Orientating (+ or −) the positions of the two daughter cells (V2a and V2b) by fate axis. (**f**) Bias in the stochastic fate decision-making according to *A*_long_ values. Receiver-operating characteristic analysis found the threshold for the shape-fate relation is *A*_long_=0.036. Red dotted line indicates 50% incidence. Scale bar, 10 μm. NS, not significant. *P<0.05 (*χ*^2^-test).

We tracked the dynamics of mitotic rounding, cell division and the fate of individual V2 cells by time-lapse confocal microscopy using *Tg(vsx1:gfp)* zebrafish embryos where green fluorescent protein (GFP) is preferentially and continuously expressed in V2 cells and their daughter cells (that is, V2a and V2b) (Supplementary Fig. 1a–d and Supplementary Movie 1). All *vsx1-gfp*^*+*^ V2 cells rounds up once (that is, mitotic rounding) before the division (Fig. 1a and Supplementary Movie 2). V2a and V2b daughter cells were distinguished by staining the cells with anti-Scl protein antibody, which stains V2b, but not V2a daughter cells (that is, V2a: *vsx1-gfp*^*+*^, Scl^−^; V2b: *vsx1-gfp*^*+*^, Scl^+^), following the time-lapse imaging of the V2 cell division (Fig. 1a).

To classify individual V2 cells according to their shapes, we designed a quantitative method to define cellular eccentricity. The three-dimensional (3D) shape of V2 cell was represented by two quantitative indices, ***D***_**asym**_ and *A*_long_. ***D***_**asym**_ is the vector indicating to which direction the cell is asymmetrically elongated (Fig. 1b, see Methods). *A*_long_ is a quantitative indicator for how much the cell is asymmetrically elongated along the long principal axis, thus is a measure of the eccentricity of the cell (Fig. 1b, see Methods). Thus, the combination of ***D***_**asym**_ and *A*_long_ defines the orientation and the degree of cellular eccentricity of each V2 cell (Fig. 1c). We first examined dynamics of V2 shapes by plotting changes of *A*_long_ over time (Supplementary Fig. 1e). *A*_long_ of each V2 cell significantly changes over time until they round up and enter into mitotic rounding phase, where *A*_long_ consistently decline (Supplementary Fig. 1e). Hence, we used V2 shapes that immediately precede mitotic rounding phase (that is, at −25 to −20 min time point relative to the time point when the cells begins to divide; this time point is indicated as ‘0 min aligned timeline' in Supplementary Fig. 1) as a putative predictor for V2 fates following the mitotic rounding and cell division. Calculation of ***D***_**asym**_ and *A*_long_ values for each V2 cell at −25 to −20 min (‘0 min aligned timeline' in Supplementary Fig. 1; that is the last unique shape that each V2 cell exhibits before entering into mitotic rounding phase) found quantitatively distinguishable cell shapes (Fig. 1d). *A*_long_ values for V2 cells range from 0 to 0.12 (Fig. 1d). Cells of perfectly symmetrical shape show *A*_long_=0, as ***D***_**asym**_=0 (that is, *C*_median_ and ***C***_**mass**_ are at the same point). *A*_long_ value for the cells of the highest degree of asymmetric shape observed in the experiment was 0.12 (Fig. 1d).

We next examined whether the cellular eccentricity of V2 cells defined by ***D***_**asym**_ (that is, the direction of the asymmetric elongation) and *A*_long_ (that is, the degree of the elongation) is correlated with the fate of V2 cells after mitotic rounding and division. On V2 cell division, two daughter cells are generated—one on the plus (+) side and the other on the minus (−) side, relative to the direction of the long axis vector, with the (+) end located at the spiky end of the cell (Fig. 1e, see Methods). We analysed which of the two fates (that is, V2a or V2b) the (+)-side daughter cell acquires following the division of each V2 cell. For V2 cells of relatively round shape (*A*_long_<0.036), the (+)-side daughter cell acquires V2a or V2b fates with virtually equal probability (Fig. 1f, upper panel—left graph). In contrast, for V2 cells of highly asymmetric shape (*A*_long_>0.036), the (+)-side daughter cell preferentially acquires V2a fate (Fig. 1f, upper panel—right graph). This result suggests a possibility that cellular eccentricity of V2 cell influences the asymmetric fate decisions even after V2 cell loses its original shape through mitotic rounding and division.

**Figure 2:**
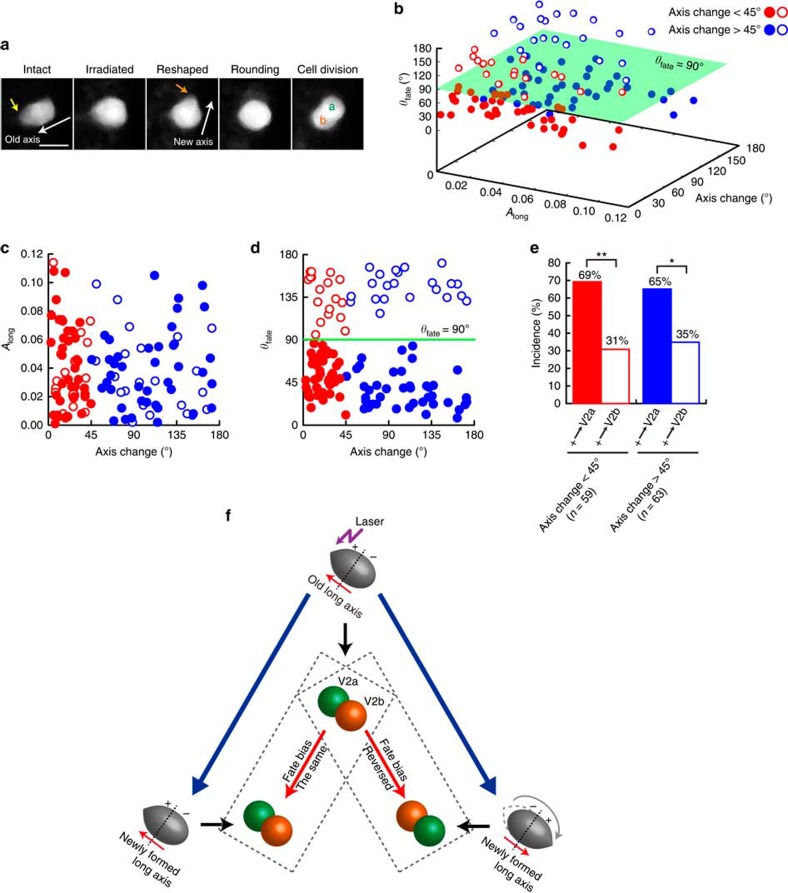
V2 cellular eccentricity biases stochastic fate decision-making. (**a**) Alteration of the direction of the asymmetric elongation of the V2 cell shape by femtosecond laser irradiation. The spiky ends of the old (yellow arrow) and new (orange arrow) axes are indicated. (**b**) *A*_long_ values, axis changes and *θ*_fate_ for individual V2 cells on femtosecond laser irradiation are shown as 3D plot. The two-dimensional plots of axis change—*A*_long_ and axis change—*θ*_fate_ are shown in **c** and **d**, respectively. Plotted are the cells with very little axis change (<45°) and *θ*_fate_ >90° (red open circle), significant axis change (>45°) and *θ*_fate_ >90° (blue open circle), little axis change (<45°) and *θ*_fate_ <90° (red closed circle) and significant axis change (>45°) and *θ*_fate_ <90° (blue closed circle). (**e**) Fates of the laser-irradiated V2 cells. (**f**) Summary. Fate decisions are made according to the newly acquired orientation of V2 cell shape asymmetry. Scale bar, 10 μm. NS, not significant. *P<0.05, **P<0.01 (*χ*^2^-test).

We investigated whether V2 cellular eccentricity indeed plays any causative role in instructing preferential acquisition of V2a fate by the (+)-side daughter cell. We addressed this question by altering the orientation of the V2 cell asymmetry by femtosecond laser irradiation (Fig. 2a). Following the femtosecond laser irradiation focused on the (+) side of the V2 cell (Fig. 2a, leftmost panel, yellow arrow), the cell shape was deformed (Fig. 2a, the second left panel), reshaped, and then ∼50% of the irradiated V2 cells successfully formed a new axis that is more than 45° difference from the old axes (Fig. 2a, the third left panel, open and closed blue circles in Fig. 2b–d). The rest formed an axis that is <45° difference from the old axis (open and closed red circles in Fig. 2b–d). All irradiated cells underwent mitotic rounding and subsequent cell division (Fig. 2a, rightmost). With the axis orientation change over 45°, the opposite-side daughter cell becomes (+) side, while the (+) side remains the same for the axis changes <45° (see Fig. 1e for the description). With all irradiated V2 cells, the daughter cells on the (+) side relatively to the new axis preferentially acquired V2a fate (Fig. 2b,e,f), hence the fate bias reverses for the cells that underwent the axis change over 45° (Fig. 2b,e,f). These results demonstrate that cellular eccentricity of V2 cells immediately before their entering into mitotic rounding phase influences their daughter cell fates. The fact that V2 cells of which a new axis remains relatively unchanged (that is, <45°) on the irradiation maintained the same fate bias (Fig. 2b,d–f), indicates that the laser irradiation itself did not influence the fate decision-making. Irradiation of neighbouring non-V2 cells had no influence on the bias in V2 fate decision-making (Supplementary Fig. 2), eliminating the possibility that a leaky irradiation effect on neighbouring non-V2 cells influences the V2 fate.

### Modelling and computational simulations

**Figure 3:**
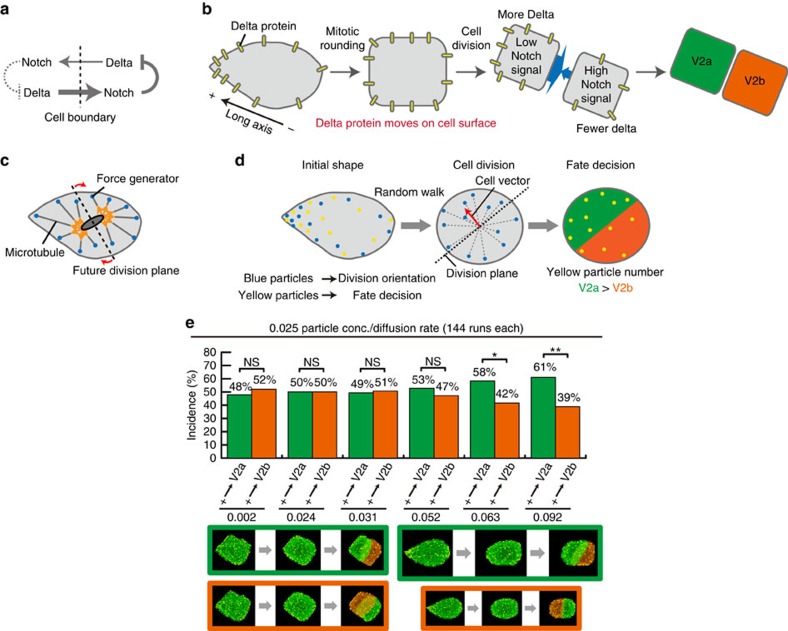
Model for shape-dependent biased fate decision-making and *in silico* validation of the model by computer simulations. (**a**) The lateral inhibition system generated by the direct Delta–Notch trans-engagement at the contacting cell surface. (**b**) A mechanistic model for the shape-dependent biased fate decision-making. (**c**) Force-generating system to drive cell division. (**d**) Integration of both cell division and fate decision-making systems into one model based on 3D CPM. (**e**) Computer simulation of the shape-dependent biased fate decision-making model. Simulation results with *in silico* cells of three relatively symmetrical (*A*_long_=0.002, 0.024 and 0.031) and three asymmetrical (*A*_long_=0.052, 0.063 and 0.092) shapes are shown. NS, not significant. *P<0.05, **P<0.01 (*χ*^2^-test). See also Supplementary Fig. 3 and Supplementary Movies 3 and 4.

The results hitherto demonstrated that V2 cellular eccentricity biases its stochastic fate decision-making despite the disappearance of such eccentricity through mitotic rounding and division. A potential mechanism underlying this ‘shape-memory' system was studied by developing a mechanistic model, which was then tested by computational simulation. A model was developed using lateral inhibition system as platform for asymmetric fate decision-making. The lateral inhibition system mediated by Delta–Notch signalling is known to form a mutually exclusive binary switch system for cell-fate specification, including the V2a/b fate specification^11^,^12^,^13^,^14^,^15^,^16^,^17^,^18^,^19^ (Fig. 3a, see Methods for further description of this signalling system). While the Delta–Notch signalling could explain why each of the two daughter cells assumes a fate different from each other, this mechanism alone cannot explain the V2 shape-dependent bias nor the stochasticity in the daughter cell-fate decision-making as found in the experiments (Fig. 1f, upper panel; and Fig. 2b).

We addressed this problem by incorporating an additional mechanism into the model—the mechanism that could account for the stochastic yet shape-dependently biased the fate decision-making of the V2 daughter cells. It was previously reported that the Delta protein is preferentially localized on the apical surface of epithelial cells in *Drosophila*^20^. Hence, we hypothesized that by localizing the Delta molecules in a polarized fashion according to the initial cellular eccentricity of V2 and making them diffuse over the cell surface as the cell rounds up during mitotic rounding, Delta could store the original shape information and bias the stochastic fate decision-making of V2 daughter cells (Fig. 3b, see Methods). In the model, Delta molecules are represented as yellow particles (Fig. 3b), and lateral inhibition and negative feedback system formed by the interaction of Delta–Notch (Fig. 3a) was described using ordinary differential equations (ODEs) as previously reported^21^, where the value of Notch was set to 0 and the value of Delta was given by the relative amounts of yellow particles in either cell as initial conditions. According to the operating principle of the lateral inhibition system, a majority of yellow particles results in lower Notch activity leading to V2a fate, whereas a minority of yellow particles results in higher Notch activity leading to V2b fate. The cell division algorithm was based on the previously reported force-generating system^2^,^3^,^22^,^23^,^24^ (Fig. 3c and see Methods). These assumptions were integrated into a mechanistic model based on the cellular Potts model (CPM)^25^ combined with an agent-based model to describe the diffusion-like behaviour of membrane-bound particles (Fig. 3d and see Methods).

The critical question to be addressed using this model is whether mitotic rounding erases the effect of the biased inductive signal formed by the polarized localization of Delta molecules (that is, erases ‘shape-memory'), or whether the cell still carries over a trace of ‘shape-memory' even after mitotic rounding, thus biasing the stochastic fate decision-making by the daughter cells. We addressed this question by running computational simulations based on this model. A total of six *in silico* V2 cells, each with increasing degree of cellular eccentricity (*A*_long_=0.002, 0.024, 0.031, 0.052, 0.063 and 0.092) were individually subjected to 144 *in silico* cell divisions. As the asymmetry (that is, *A*_long_) increases, the *in silico* V2 cells exhibit significant bias towards the V2 fate acquisition by the (+)-side daughter cell (Fig. 3e and Supplementary Movies 3 and 4), quantitatively matching the experimentally observed probability distribution for the shape-fate relation (Fig. 3e, compare with Fig. 1f). Division of the *in silico* V2 cells followed the long-axis rule (Supplementary Fig. 3f,g)^1^,^2^,^3^, as the real V2 cells do *in vivo* (Supplementary Fig. 3h).

**Figure 4:**
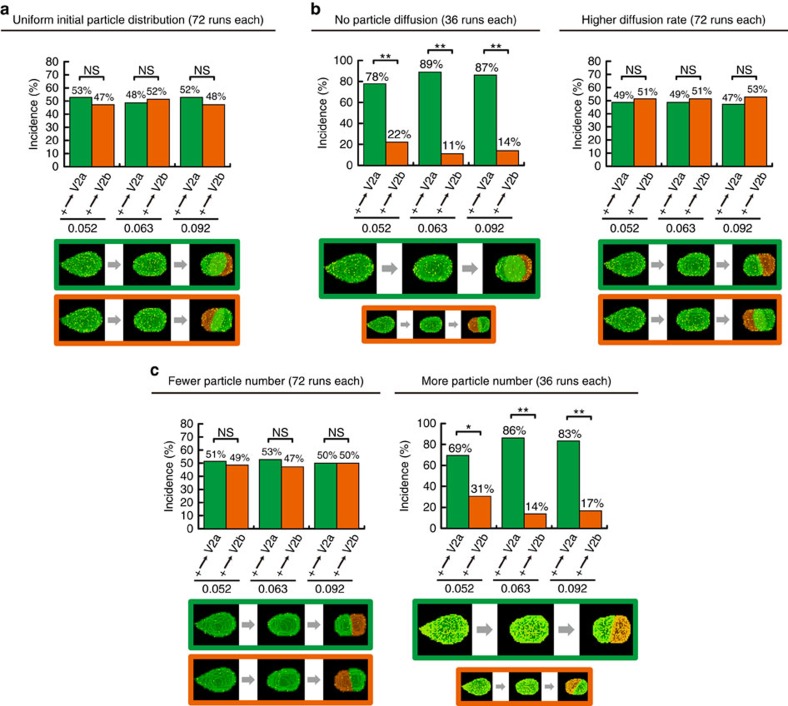
Simulating the effects of Delta molecule dynamics on the bias. (**a**) No bias in the fate decision-making with no initial polarized localization of Delta particles, despite the asymmetrical shape. Simulation results with three asymmetrical (*A*_long_=0.052, 0.063 and 0.092) shapes are shown. (**b**) Diffusion rate-dependent bias in the fate decision-making of the *in silico* cells of asymmetrical shapes. Higher diffusion rate: four lattice site length per mcs. Simulation results with three asymmetrical (*A*_long_=0.052, 0.063 and 0.092) shapes are shown. (**c**) Weaker and stronger biases in the fate decision-making with fewer (0.0025 particle concentration (conc.)/diffusion rate) or more Delta particles (0.25 particle conc./diffusion rate), respectively. Simulation results with three asymmetrical (*A*_long_=0.052, 0.063 and 0.092) shapes are shown. NS, not significant, *P<0.05; **P<0.01 (*χ*^2^-test).

The model was validated *in silico* by examining its critical parameters. In the model, the fate bias must be critically dependent on the initial condition of the polarized Delta localization (Fig. 3b). Thus, we ran the simulation with *in silico* V2 cells where Delta particles were uniformly distributed (Fig. 4a). Virtually no fate bias was found when Delta particles (that is, yellow particles) were uniformly distributed in the *in silico* V2 cells despite their highly asymmetric shape (Fig. 4a), validating that the criticalness of the shape-dependent polarization of Delta localization for the model. Another critical parameter is the diffusion rate of Delta particles (Fig. 3b). The effects of the diffusion rate of Delta particles relative to the V2 cell mitotic rounding and division was evaluated by performing simulations (Fig. 4b). The simulations with no diffusion or higher diffusion rates (four lattice site length per Monte Carlo step (mcs)) resulted in more bias (that is, nearly deterministic; Fig. 4b, left panel; compare with Fig. 1f) or less bias (Fig. 4b, right panel; compare with Fig. 1f), respectively. These simulation results demonstrate that the diffusion rate of Delta molecules as the cell rounds up is critical for the experimentally observed degree of bias in the fate decision-making. Next, we examined the third critical parameter of the model: the number (that is, abundance) of Delta molecules (Fig. 3b). This was tested by running the simulations with varying number of Delta particles (Fig. 4c). The *in silico* V2 cells with the reduced Delta level (0.0025 particle conc./diffusion rate in Fig. 4c, left panel, as compared with 0.025 particle conc./diffusion rate in Fig. 3e) exhibited virtually no bias (Fig. 4c, left panel; compare with Fig. 3e). The *in silico* V2 cells with the increased Delta level (0.25 particle conc./diffusion rate in Fig. 4c, right panel, as compared with 0.025 particle conc./diffusion rate in Fig. 3e) showed significantly enhanced bias (Fig. 4c, right panel; compare with Fig. 3e). The results from these *in silico* validation experiments are all in agreement with the mechanistic model shown in Fig. 3b.

### Experimental validation of the *in silico* mechanistic model

**Figure 5:**
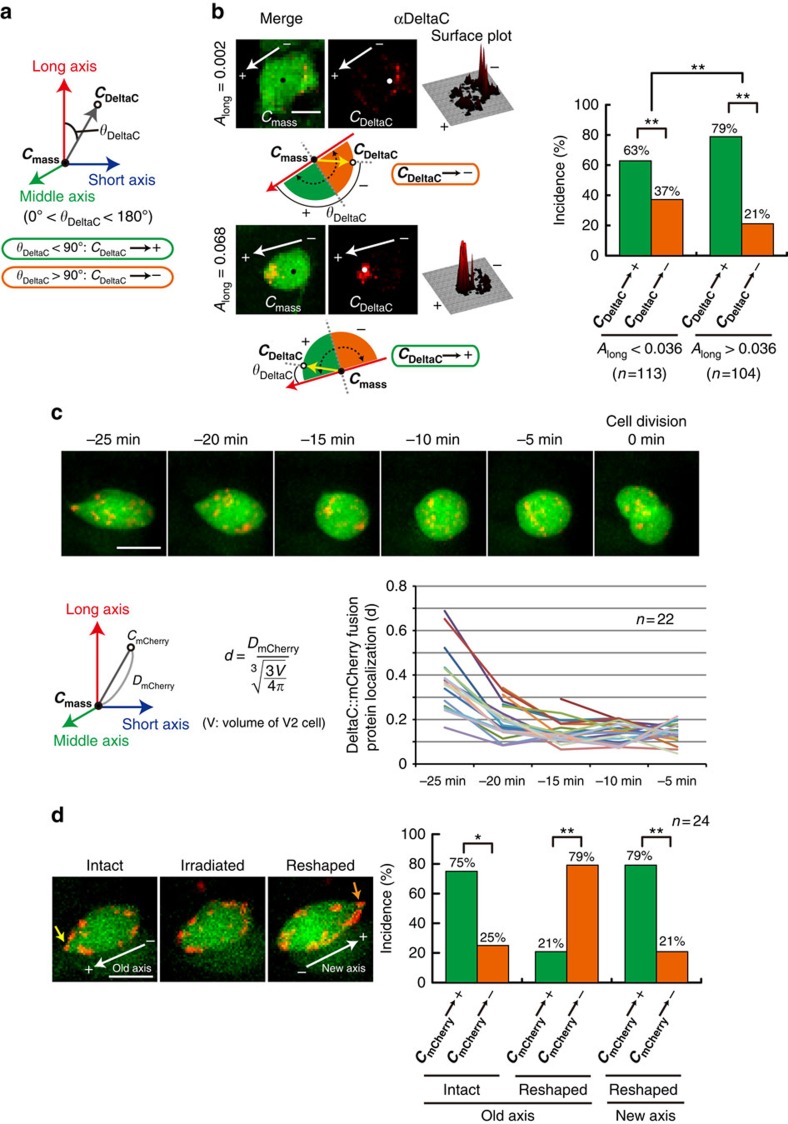
V2 cell eccentricity polarizes the localization of DeltaC protein. (**a**) Quantitative measurement of DeltaC protein distribution. (**b**) Polarized localization of DeltaC protein on the (+) side of V2 cell. (**c**) Diffusion-like behaviour of the initially polarized DeltaC::mCherry fusion protein during mitotic cell rounding. (**d**) Translocation of DeltaC::mCherry fusion protein on changes of the direction of the asymmetric elongation of V2 cell shape. The spiky ends of the old (yellow arrow) and new (orange arrow) axes are indicated. Irradiation at the spiky ends of the old axis (yellow arrow) altered the asymmetry orientation generating the new axis. Biased localization of the DeltaC::mCherry fusion protein is shifted according to the newly acquired orientation of V2 cell shape asymmetry. *P<0.05, **P<0.01 (*χ*^2^-test). Scale bars, 5 μm (**b**), 10 μm (**c**,**d**). See also Supplementary Fig. 4 and Supplementary Movies 5 and 6.

The biological relevance of the mechanism for the ‘shape-memory' system predicted by modelling and simulation was examined by experimentally validating the model. First, we determined which Delta protein is expressed in V2 cells. In zebrafish, there are four known Delta proteins, DeltaA, DeltaB, DeltaC and DeltaD (ref. 26). Immunostaining the zebrafish embryo with antibody to each Delta protein found that DeltaC, but not Delta A or D, is expressed in V2 cells (Supplementary Fig. 4). The cell-surface localization of DeltaC protein molecules was confirmed by examining optical serial sections of GFP^+^ V2 cells stained with anti-DeltaC antibody using confocal laser microscopy (Supplementary Fig. 5). We next investigated whether DeltaC protein exhibits preferential localization on the (+) side of V2 cell, as determined critical for the system by the simulation (Figs 3e and 4a). The localization of DeltaC protein in V2 cells was analysed by calculating ***C***_**DeltaC**_, the centre of mass of all the DeltaC protein positive staining signals (Fig. 5a). The result indeed shows the polarized distribution of DeltaC protein towards the (+) side of the V2 cell before mitotic rounding (Fig. 5b). The bias was stronger for the V2 cells with higher degree of eccentricity—that is, larger *A*_long_ values (Fig. 5b). Diffusion-like behaviour of Delta proteins as V2 cell rounds up, as determined critical for the system by the simulation (Fig. 4b), was next studied by live imaging of DeltaC::mCherry fusion protein (Supplementary Fig. 6a,b and see Methods) expressed in V2 cells. Following the localization of DeltaC::mCherry fusion protein at the series of time points (−25, −20, −15, −10 and −5 min) before division (0 min is the time when the cell began dividing) indicated their diffusion-like behaviour (Fig. 5c, upper panel; and Supplementary Movie 5). Such DeltaC::mCherry fusion protein behaviour was quantitatively analysed by calculating the distance (*D*_mCherry_) of ***C***_**mCherry**_ from the ***C***_**mass**_ (Fig. 5c, lower left panel). As the cell rounds up over time, we normalized *D*_mCherry_ by dividing it with a volume factor and obtained the value *d*, an indicator for how far the overall DeltaC::mCherry proteins are localized away from the centre of V2 cell—that is, the higher *d* values indicate more polarized localization and the less *d* values indicate less polarized thus more uniform localization (Fig. 5c, middle formula). Examining a total of 22 cells at each time point and plotting *d* values for each cell demonstrate that the localization of DeltaC::mCherry proteins shifts from the polarized to uniform patterns over time as each cell rounds up through mitotic rounding (Fig. 5c, lower right panel). Z-sectioning of the images shows the cell-surface localization of DeltaC::mCherry proteins (Supplementary Fig. 6c), indicating that the diffusion-like behaviour of DeltaC::mCherry fusion proteins occurs at the cell surface. The causal relation between V2 cellular eccentricity and the Delta localization was evaluated by artificially changing the V2 cell shape by femtosecond laser irradiation (Fig. 5d, left panel). DeltaC::mCherry fusion protein was expressed in V2 cells and its localization was examined following the shape-change induced by femtosecond laser irradiation (Fig. 5d, left panel). The result showed that the DeltaC::mCherry protein localization was shifted towards the newly generated (+) side (Fig. 5d, right panel, and Supplementary Movie 6), demonstrating that the V2 cell shape is causal in localizing DeltaC protein. V2 cells that failed to generate a new axis on the irradiation maintain the same polarized localization of DeltaC::mCherry proteins (Supplementary Fig. 7), indicating that the laser irradiation itself does not influence the DeltaC::mCherry localization. Taken together, the results shown in Fig. 5 establish the causal relation between the V2 cell eccentricity and DeltaC localization.

**Figure 6:**
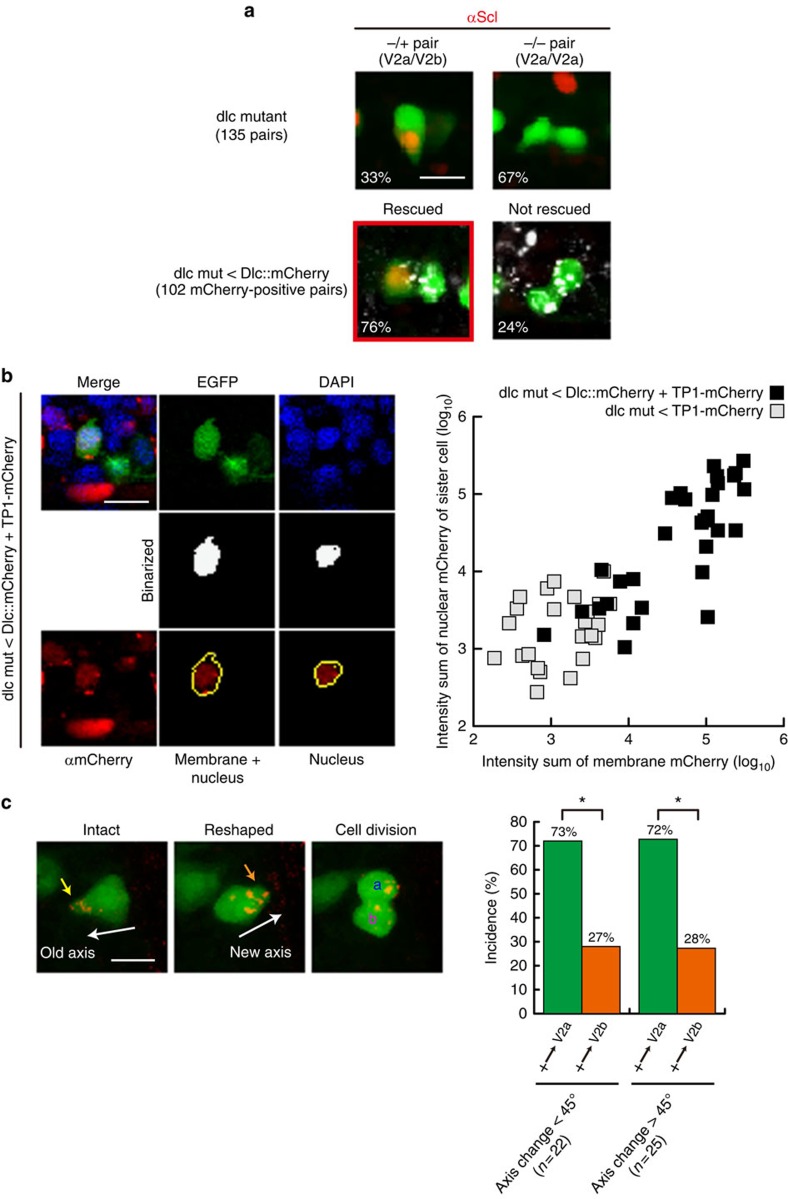
DeltaC is an essential mediator of the ‘shape-memory' system. (**a**) Failure of V2a/V2b fate decision-making in DeltaC-deficient V2 cell and its rescue by DeltaC re-expression. (**b**) Reduced Notch-signalling activity in DeltaC-deficient V2 cell and its rescue by DeltaC re-expression. See Methods for the details of this assay system and the data analyses. (**c**) Rescue of the biased fate decision-making accompanied by the biased DeltaC protein localization in the deltaC-deficient V2 cells by re-expressing DeltaC. The spiky ends of the old (yellow arrow) and new (orange arrow) axes are indicated. *P<0.05 (*χ*^2^-test). Scale bars, 10 μm.

**Figure 7:**
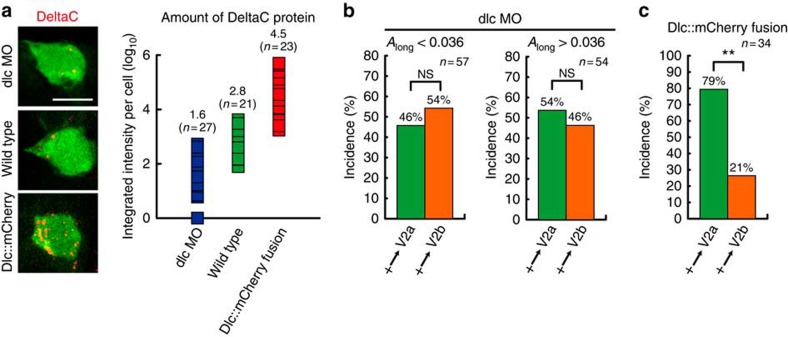
The bias is dependent on the abundance of DeltaC molecules. (**a**) Altered DeltaC protein levels by loss- and gain-of-functions. Scale bar, 10 μm. (**b**) Attenuation of the bias by the reduced DeltaC protein level. (**c**) Enhancement of the bias by the increased DeltaC protein level. NS, not significant. **P<0.01 (*χ*^2^-test). See also Supplementary Fig. 5.

Next, we experimentally evaluated whether DeltaC and its behaviour indeed causally bias the stochastic cell-fate decision-making (Figs 6 and 7). First, we examined the asymmetric fate decision-making of DeltaC-deficient V2 daughter cells in *deltaC* mutant (Fig. 6a). The *deltaC* mutant is viable and exhibits normal V2 cell formation and division despite the lack of DeltaC, however, both daughter cells assume V2a fate (Fig. 6a, upper panels). The lack of the asymmetric fate decisions of the V2 daughter cells were rescued by re-expressing DeltaC proteins in the *deltaC*-deficient V2 cells in the mutant (Fig. 6a, lower panels). In this gain-of-function experiment, DeltaC was re-expressed in V2 cells using *deltaC* BAC to recapitulates the endogenous DeltaC expression pattern (Fig. 6a, lower panel). The lack of DeltaC in the mutant and the re-expression of DeltaC resulted in the reduced and regaining of the Notch-signalling activity, respectively, as demonstrated by measuring the Notch-signal reporter (TP1-mCherry) activity in V2 cells (Fig. 6b, see Methods for the details of this assay system and the data analyses). The causal and V2 cell intrinsic role of the polarized localization of DeltaC in biasing the V2 cell fate was further validated by re-expressing DeltaC in the shape-manipulated *deltaC* mutant V2 cell (Fig. 6c). The re-expressed DeltaC protein in the *deltaC*-deficient V2 cell exhibited the expected polarized localization of DeltaC protein at the spiky end of V2 cell (Fig. 6a,c), which was accompanied by correctly re-gaining of the biased fate determination (Fig. 6a,c). Furthermore, the alteration of the orientation of the spiky end by femtosecond laser irradiation resulted in shifting the re-expressed DeltaC localization towards the newly generated spiky end and in the fate bias according to the new axis orientation (Fig. 6c).

The DeltaC-dependence of the bias in the fate decision-making was further validated by varying the abundance of Delta molecules using loss-of-function and gain-of-function approaches (Fig. 7). The loss-of-function and gain-of-function was accomplished by morpholino-mediated knockdown of DeltaC and overexpressing DeltaC::mCherry, respectively. The levels of the increase and the reduction of DeltaC protein were quantified by measuring the intensities of DeltaC staining in V2 cells (Fig. 7a). This analysis indicated that the gain-of-function increased the DeltaC protein level by ∼20-fold and the loss-of-function decreased its level by ∼10-fold (Fig. 7a). The morpholino-mediated knockdown attenuated the bias (Fig. 7b, compare with Fig. 1e), and the gain-of-function enhanced the bias (Fig. 7c, compare with Fig. 1e). These experimental results validate the predictions made by the computational simulation (Fig. 4c), validating the importance of DeltaC abundance in biasing the fate. Thus, the experimental results shown in Figs 6 and 7 establish the causal relation between DeltaC and the biased fate decision-making, validating the biological relevance of the model.

## Discussion

**Figure 8:**
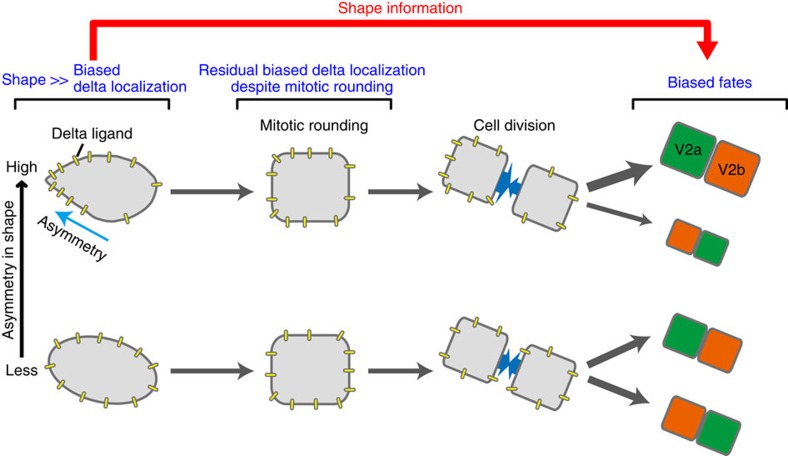
Schematic diagram of the ‘shape-memory' system medicated by the biased DeltaC protein localization. Polarized DeltaC localization caused by V2 cell eccentricity biases its stochastic fate decision-making even after mitotic rounding.

The importance of the interdependence of cell shape and cell function in biology has been recognized for centuries and has precipitated numerous intense investigations. Most recently, theoretical and mathematical approaches have been combined together with experimental studies to gain quantitative insights into the dynamics of shape–function relationship^2^,^3^,^5^,^8^,^9^. While all of these data clearly indicated that cell shape critically instructs cellular function and/or fate, such shape-dependent influence persists only transiently, thus disappearing on the original shape changes or is lost. However, cells dynamically change their shapes *in vivo* where multiple and complex intrinsic and extrinsic factors are constantly influencing cell shape. Hence, it is important to determine whether past cell shape information remains influential on future cell function and/or fate even after the cell changes the shape. In this paper, by integrating both experimental and computational approaches, we demonstrate that the past shape information remains influential on the future cell fate even after the dynamic changes of the past shape (Fig. 8). We also show that such ‘shape-memory' system is mediated by the shape-dependent biased localization of DeltaC protein (Fig. 8).

We addressed this question with an asymmetric fate decision-making system in developing zebrafish embryo. We first developed a method using two parameters, ***D***_**asym**_ and *A*_long_, that quantify the orientation and the degree of cellular eccentricity, respectively (Fig. 1b). This method enabled us to find a correlation between eccentricity and fate of individual V2 cells (Fig. 1f). Previously, it was reported that the surface-to-volume (SAV) ratio of the cells contributes to cell signalling and/or function^27^,^28^. Hence, we calculated SAV ratios of individual V2 cells, but found no correlations between their SAV ratios and fates (Supplementary Fig. 8). This may reflect the fact that V2 cells *in vivo* vary in size, in contrast to cells in culture where the cell size is relatively uniform^27^,^28^.

Using quantitative live imaging and femtosecond laser-mediated shape manipulation, we demonstrate that V2 progenitor cell shape elicits a causative influence on biasing the stochastic fate decision-making of the daughter cells even after V2 cell loses the original geometry through mitotic rounding and division (Figs 1f and 2b,d–f). On the basis of these findings, we developed a model that was then tested by computer simulation to gain insights into the mechanism for this ‘shape-memory' system (Figs 3 and 4). The simulation studies suggested that if Delta protein is more abundantly localized towards the spiky of the elongated V2 cell, the daughter cell divided from the spiky side receives subtle but sufficiently more Delta molecules than the other daughter cell after cell division despite the cell-surface diffusion of Delta molecules through mitotic rounding. This biases the daughter cells divided from the spiky side towards V2a fate (Fig. 3b,e). The simulation with varying parameters suggested that the shape-dependent polarized localization of Delta protein is critical in biasing the stochastic fate decision-making of the daughter cells (Fig. 4a). It also indicated that no diffusion or higher diffusion rates of Delta molecules result in excessive or no bias, respectively (Fig. 4b). With no diffusion, the polarized Delta protein localization pattern remains even through mitotic rounding, hence, the daughter cells divided from the spiky side almost always receive more Delta proteins, acquiring V2a fate. In contrast, with higher diffusion rate, Delta molecules diffuse more quickly relative during mitotic round and as a result they are distributed uniformly in V2 cell, hence both daughter cells acquire V2a and V2b with equal probability. The simulation also showed that the fewer or more Delta molecules cause no or excessive bias, respectively (Fig. 4c). With fewer Delta molecules, it takes less time for the polarized Delta protein localization pattern to disappear via diffusion, causing both daughter cells to receive slightly more or less number of Delta molecules with equal probability, thus leading to no bias in acquiring V2a or V2b fates. In contrast, with more Delta molecules, the polarized Delta localization pattern is more robust, causing the daughter cell divided from the spiky side to receive more Delta molecules with higher probability, thus leading to more bias in her V2a fate acquisition. All of these simulation results supports the idea that the shape-dependent Delta protein localization is an origin of the ‘shape-memory' system, and diffusion rate and abundance of Delta molecules quantitatively impact which side of the daughter cells receive more Delta molecules at the time of V2 progenitor cell division, hence influencing the degree of the bias.

These predictions were experimentally validated. We show that endogenous DeltaC proteins are more enriched towards the spiky end of V2 cells (Fig. 5a), and the exogenously expressed reporter DeltaC (that is, DeltaC::mCherry) localization shifts towards the newly generated spiky end of V2 cells on shape changes induced by the laser irradiation (Fig. 5d). Using *deltaC*-deficient mutant zebrafish, where all V2 daughter cells acquire V2a fates, we also demonstrate that the re-expressed DeltaC protein in the *deltaC*-deficient V2 cells exhibits the expected biased localization towards the spiky end of V2 cells (Fig. 6a,c). Such biased localization of exogenously introduced DeltaC protein in the *deltaC*-deficient V2 cells biases the binary (that is, V2a or V2b) fate decision-making of V2 (Fig. 6c) in a shape-dependent manner. In this series of genetic rescue experiments, the re-expression of DeltaC is in V2 cells, but not in the neighbouring non-V2 cells (Fig. 6a,c), as the expression is mediated by V2 BAC vector. Furthermore, no influence of the laser irradiation-mediated damaging of the neighbouring non-V2 cells on the shape-dependent biased V2 cell-fate specification was found (Supplementary Fig. 2). These results support the notion that the role of the shape-dependent DeltaC is causal and V2 cell intrinsic (that is, not mediated via neighbouring non-V2 cells) in the biased fate decision-making.

What is the mechanism for the shape-dependent DeltaC protein localization? We show shape-dependently biased DeltaC protein localization on the surface of V2 cells (Figs 5 and 6). It has been shown that localized protein synthesis from the mRNA located at a unique subcellular domain contributes to the unique protein localization in the cell^29^. In the V2 cells, *deltaC* mRNA is uniformly distributed in the cytoplasm (Supplementary Fig. 9), suggesting that the biased DeltaC protein localization is due to shape-dependent translocation of DeltaC proteins, rather than due to the localized DeltaC protein synthesis.

Previously, it has been shown that membrane curvature is a critical determinant of cell shape information^8^. However, cytoskeletal arrangement and/or mechanical feature such as cellular tension also critically contribute to subcellular protein localization^30^,^31^,^32^. While actin fibre density within V2 cells is relatively uniformly distributed (Supplementary Fig. 10), interfering with actin polymerization or ATPase activity of non-muscle type myosin II, but not tubulin polymerization, results in the disintegration of the shape-dependent biased DeltaC protein localization (Supplementary Fig. 11). These results suggest a role of actin cytoskeleton and cellular tension in inducing and/or maintaining the shape-dependent DeltaC protein localization.

Previous simulation study accompanied by experimental validation data indicated that a role of cAMP/PKA/MAPK signalling in transduction of cell geometry information^28^. We examined a role of this signalling pathway in the shape-dependent DeltaC localization and found inhibitors of this signalling pathway had no influence on the biased DeltaC localization (Supplementary Fig. 12).

In our model (Fig. 3b), as validated by computational simulation (Figs 3e and 4), the behaviour of Delta protein alone is sufficient to explain the ‘shape-memory' system of V2 cells. Are there any other factors that critically contribute to this system? It has been reported that secreted molecules induce asymmetric stem cell division^33^. They show that a localized Wnt signal orients asymmetric stem cell division *in vitro*, suggesting a possibility that such secreted molecule can provide an instructive signal for asymmetric cell division.

It has been previously shown that cells undergoing mitotic rounding exhibit retraction fibres that anchor the cells onto the substratum^24^,^34^, suggesting a role of neighbouring cells or substratum on the fate or behaviour of the cells undergoing mitotic rounding. However, in our *in vivo* system of V2 cells, we found no such retraction fibres with V2 cells undergoing mitotic rounding (Supplementary Fig. 13).

V2 cells are surrounded by non-V2 cells, but laser-mediated damaging of these non-V2 cells does not influence the biased fate decision-making of V2 cells (Supplementary Fig. 2). Our simulation and experimental results show that the bias in the ‘shape-memory' system is critically dependent on the abundance of Delta molecules (Figs 4c and 5b). While the abundance of Delta protein is primarily regulated at the level of transcription^12^, the activity of Delta–Notch signalling is also modulated by ubiquitylation^35^,^36^ and endocytosis^37^. Therefore, contributions of other factors to the ‘shape-memory' system cannot be excluded. However, our genetic mutation and rescue experiments (Fig. 6) demonstrate that DeltaC is absolutely essential for the ‘shape-memory' system of V2 cells. Furthermore, our model and computational simulation studies show that the lateral-inhibition system enables the cells to exploit the subtle differences in the number of fate-inducing molecules like Delta to make mutually exclusive binary fate decisions (Fig. 3a). Hence, DeltaC, if not the only factor, is an essential factor for mediating the ‘shape-memory' system of V2 cells. In fact, several other signalling molecules have been shown to exhibit cell-polarity-dependent localizations^38^,^39^,^40^. It would be interesting if these signalling molecules such as PKC, PAR-3 and/or Cdc42, together with DeltaC, participate in the shape-dependent biasing process of V2 cell-fate decision-making.

Our computer simulation studies indicate that in addition to the shape-dependent polarized DeltaC localization, both diffusion rates and the abundance of DeltaC molecules quantitatively influence the degree of the fate bias (Fig. 4b,c). While the known physiological ranges of diffusion rate and the molecular abundance of cellular proteins were taken into account for the simulation, the exact quantitative information on the diffusion rate and the abundance of DeltaC molecules in V2 cells in zebrafish is lacking. Nor do we know the effects of Notch ligation to DeltaC molecules and/or clustering of DeltaC molecules on their diffusion rates. Addressing such questions using single-molecule imaging techniques could further validate our model in the future.

In this study we show that shape information not only has transient effect as previously shown^8^ but also could be stored as a ‘memory' for an extended period of time and influences the future cell fate despite the loss of the past geometric information of the cell. It will be interesting to investigate other types of information storage and retrieval systems for shape that cells could exploit to establish and/or control their future function and fate according to their past shape information. Such studies facilitate our understanding of shape–function interdependence and the underlying information storage, processing and retrieval systems in the cell. Further understanding of such mechanisms could also improve the use of cell geometry as predictive indices for the future function and fate of the cells, which may find useful applications to pathologic and/or clinical diagnosis.

## Methods

### Zebrafish breeding and maintenance

Zebrafish fertilized eggs were collected in Egg raising buffer (0.06% artificial marine salt supplemented with 0.0002% methylene blue) and were raised at 23–31 °C. Staging of embryos were according to Kimmel *et al*.^41^. The transgenic line, *TgBAC(vsx1:GFP)*^*nns5*^, and *deltaC*^*tit446*^ are as previously described^11^,^42^. All animal protocols were approved by the Animal Care and Use Committee of Nara Institute of Science and Technology (Permit Number: 1234) and Advanced Telecommunications Research Institute International (Permit Number: A1403).

### Time-lapse microscopy

*TgBAC(vsx1:GFP)* embryos at 14-somite stage (16 h post fertilization (h.p.f.)) were mixed with 0.35–0.4% low-melting-point agarose, then put into 0.5-mm width slit on 1% agarose-coated glass-bottomed Petri dishes to fix the orientation of embryos. To stop spontaneous movements, the 0.003% Tricaine solution was added. Time-lapse imaging by confocal microscopy was performed with × 20 dry (numerical aperture=0.8) objective lens mounted on Zeiss LSM710 (Zeiss, Germany) with an incubator to maintain embryos at 25 °C. Z image stacks of neural tube from the third to the eighth somite levels were captured with an optical slice thickness at 5-min intervals. After 10 h of observation, embryos were fixed and processed for immunohistochemistry.

### Immunohistochemistry

Embryos were fixed in 4% paraformaldehyde in phosphate-buffered saline (PBS) for 2–3 h at room temperature or overnight at 4 °C. Whole-mount antibody staining was performed using the following antibodies: anti-DeltaA (18D2, ZIRC) (1:50); anti-DeltaC (zdc2, Abcam, UK) (1:500); anti-DeltaD (zdd2, Abcam, UK) (1:500); anti-Scl (ref. 43) (1:50); anti-mCherry (ab167453, Abcam, UK) (1:500); and anti-RFP (8D6, MBL, Japan) (1:500). Goat anti-mouse IgG Alexa568 (A-11031, Thermo Fisher Scientific, USA) (1:1,000) and goat anti-rabbit IgG Alexa546 (A-11035, Thermo Fisher Scientific, USA) (1:1,000) were used as the secondary antibodies. The Histofine Simple Stain MAX PO (R) (Nichirei Bioscience, Japan) and the TSA Cyanine 5 System (PerkinElmer, USA) were also used for Scl detection.

### Quantitative measurements of cell shapes

The basic idea of our method is to indicate the direction and the degree of the asymmetric elongation of V2 cell shape by two indices ***D***_**asym**_ (vector) and *A*_long_, respectively (Fig. 1b). ***D***_**asym**_ is a vector that projects from ***C***_**mass**_ (the centre of mass of the V2 cell) to *C*_median_ (the centre of the bounding box enclosing the V2 shape). The direction of the vector indicates to which direction the V2 cell is asymmetrically elongated (Fig. 1b). The scalar (*D*_asym_) of the vector ***D***_**asym**_ could represent the degree of the asymmetric elongation. However, as the cell volume varies from one cell to another, we invented a new index *A*_long_, to indicate the ‘volume-normalized' extent of the asymmetric elongation of V2 cells (Fig. 1b). The detailed mathematical methods of the calculations for ***D***_**asym**_ and *A*_long_, are described as follows.

To quantitatively analyse cell shape, we made a 3D reconstruction of the V2 cells based on the confocal images. 3D median filter was first applied to fluorescent images of V2 cells. Image binarization was performed with discriminant analysis method or with manually determined threshold values. The principal axes of the moments of inertia were calculated for each V2 cell. The inertia tensor ***I*** is defined as below:


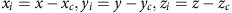



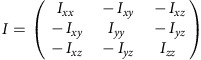



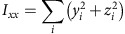



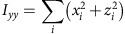



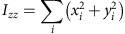



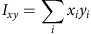



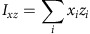



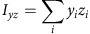


where (*x*_*c*_, *y*_*c*_, *z*_*c*_) is the coordinate of the centre of mass of the cell (***C***_**mass**_). Eigenvectors ***e*** and eigenvalues *λ* satisfy an equation below:


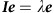


Then, we obtain


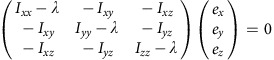



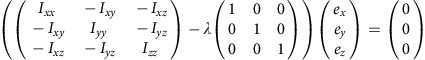


Therefore,


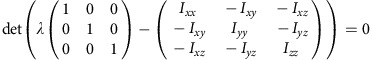


Solving above simultaneous equations, we obtain the eigenvectors and eigenvalues of the inertia tensor of 3D reconstructed V2 cell shape. The eigenvector with the biggest eigenvalue (***e***_**short**_) is the directional vector of short axis. The eigenvector with the smallest eigenvalue (***e***_**long**_) is the directional vector of long axis. The eigenvector with the middle eigenvalue (***e***_**mid**_) is of middle axis.

We calculate three inner products for every voxel in the object:


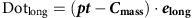



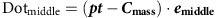



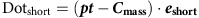


where ***pt*** is voxel coordinates in the object.

Then we calculate the principal axis-aligned minimum bounding box for a given V2 cell shape and obtain the vector, ***D***_**asym**_ from the ***C***_**mass**_ to the centre of the bounding box (*C*_median_):


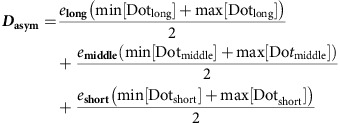


If ***D***_**asym**_·***e***_**long**_<0, ***a***_**long**_=−***e***_**long**_. Otherwise, ***a***_**long**_=***e***_**long**_.

If ***D***_**asym**_·***e***_**middle**_<0, ***a***_**middle**_=−***e***_**middle**_. Otherwise, ***a***_**middle**_=***e***_**middle**_.

If ***D***_**asym**_·***e***_**short**_<0, ***a***_**short**_=−***e***_**short**_. Otherwise, ***a***_**short**_=***e***_**short**_.

We re-defined the directions of the principal axes along three unit vectors, ***a***_**long**_, ***a***_**middle**_ and ***a***_**short**_.

Then *D*_long_ is given as below:


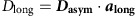


To normalize *D*_long_ values, the value is divided by the radius of a sphere with the volume (*V*) equal to that of V2 cell.


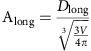


The process is semi-automated. The scripts can be made available on request.

### Orienting the daughter cell fates

The two daughter cells were distinguished by their positions relative to the direction (− to + direction) of the long axis as an index, *θ*_fate_. The detailed mathematical calculation methods are described as follows: 3D reconstruction of images was performed using the ImageJ^44^ software and cell fates were traced by direct observation of time-lapse images. The longest principal axis of the moments of inertia of a dividing V2 cell was calculated as the cell division axis. The direction of the unit vector of the cell division axis (***a***_**CD**_) was determined to be from the daughter cell, which acquired the V2b fate to the other daughter cell, which chose the V2a fate, and cell division axis (***a***_**CD**_) was referred to as ‘fate axis' vector. The angles between principal axes and fate axis (a.k.a. division axis) are given as below:


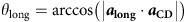



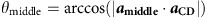



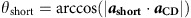



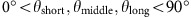


The daughter cell fate is represented according to the angle (*θ*_fate_) between two vectors, ***a***_**long**_ and ***a***_**CD**_.


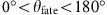


If cos *θ*_fate_=***a***_**long**_·***a***_**CD**_>0, the daughter cell on the plus (+) side of the long axis is the V2a.

If cos *θ*_fate_=***a***_**long**_·***a***_**CD**_<0, the daughter cell on the plus (+) side of the long axis is the V2b.

### Quantification of dynamic localization of delta protein

Plugins, 3D object counter^45^ and 3D Roi manager^46^ were used to calculate the sum of all intensity values of fluorescent staining for DeltaC protein (*B*_sum_) and the intensity centre, ***C***_**DeltaC**_ and ***C***_**mCherry**_ in each V2 cell:


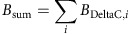



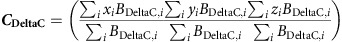



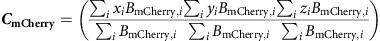


where *B*_DeltaC_ and *B*_mCherry_ are the brightness of voxels in fluorescent images for DeltaC protein and DeltaC::mCherry fusion protein, respectively. Then we obtain


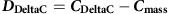



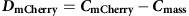


If ***a***_**long**_·***D***_**DeltaC**_>0, DeltaC protein is localized on the plus (+) side along the long axis of V2 cell.

If ***a***_**long**_·***D***_**mCherry**_>0, DeltaC::mCherry protein is localized on the plus (+) side along the long axis of V2 cell. The distance between the centre of mass of the cell and the intensity centre of DeltaC::mCherry fusion protein (*D*_mCherry_) is divided by the radius of a sphere with the volume (*V*) equal to that of the V2 cell to quantify the movement of DeltaC::mCherry fusion protein.


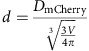


### Near-infrared femtosecond (NIR-fs) laser manipulation

*TgBAC(vsx1:GFP)* embryos at 16-somite stage (17 h.p.f.) were mounted as described above. NIR-fs laser pulses from Ti:sapphire femtosecond laser systems (Mai Tai DeepSee, 720 nm) were introduced to FV1000MPE or FVMPE-RS multiphoton laser scanning microscopes (Olympus, Japan). The pulse energy was tuned not to kill V2 progenitor cells but to modify their shapes. Laser pulses were focused on a spiky end of a single V2 progenitor cell through a × 25 water immersion lens (Olympus, XLPlan N, numerical aperture=1.05) equipped on the microscopes. The duration of the irradiation for each cell was 50 ms. Modification of the spike after the stress loading was checked with eyes in live cell images. After the irradiation of several V2 cells, embryos were maintained at 25 °C and *Z*-stacks were collected every 5 min for 2 h. Then embryos were fixed and processed for immunohistochemistry. Of 223 shape-modified V2 cells, we analysed 122 cells, which successfully proceeded to the division within 30 min after the laser irradiation. Those that did not divide within 30 min after the laser irradiation continued to change the long-axis orientations instead of entering into mitotic rounding. This indicates that they are at much earlier stages than at −20 min time point where their cellular eccentricity was used as our fate predictor in this study.

### Cell border visualization

Border between the cells were visualized by expressing mCherry::CAAX fusion protein. In the pCS2P+ vector, mCherry cDNA was fused to CAAX, and the RNA was transcribed and injected into the embryos.

### Injection of morpholino antisense oligonucleotide and RNA

Morpholino oligonucleotides (MO, Gene Tools, USA) and capped RNA synthesized with mMESSAGE mMACHINE (Ambion, USA) were injected into one- to two-cell-stage embryos. MO sequences and the injected doses were as follows: *deltaC* MO, 5′-GCACGTTAATAAAACACGAGCCATC-3′ (ref. 47), 5 ng for MO and 0.5 ng for RNA.

### BAC engineering and transgenesis

Two BACs were generated by modification of *deltaC* BAC clone DKEY-46I14 using the Red/ET recombination system (Gene Bridges, Germany)^48^. Homologous recombination was performed following the manufacture's instruction. For *Tol2* transposon-mediated BAC transgenesis, the iTol2-amp cassette^49^ was introduced into the backbone (pIndigoBAC-536) of the *deltaC* BAC. For the generation of mCherry/SV40 poly(A) signal/FRT-Kan-FRT cassette, the sequence encoding GFP in pBSK(+)-GFP-FRT-kan-FRT^49^ was replaced by the sequence encoding mCherry. To introduce the mCherry sequence into the *deltaC* locus, the mCherry/SV40 poly(A) signal/FRT-Kan-FRT cassette was amplified by PCR using the following primer pairs, encoding left and right homology arms. The sequence homologous to the PCR template is underlined. Lower-case letters represent Kozak sequence and the start codon. 5′-GGACTACTCTCACAGTCTGCTATCGTTCAGTAGCAGACAAGAAGGCAAAGgccaccatgGTGAGCAAGGGCGAGGAGGATAAC-3′ and 5′-CTCACCAAATGCGATGATATCAAAATAAAAAAGCACGTTAATAAAACACGCCGCGTGTAGGCTGGAGCTGCTTC-3′ for *deltaC:mCherry*; 5′-CAAAGAACGTGCATCAATGCCATTTTTTCCTTTCTGTCCTTCCTCAGGTAGTGAGCAAGGGCGAGGAGGATAAC-3′ and 5′-TTGTGGTCAGGCCCACTGGTGTTTGAGGTGCTCCAGATTGAAGAATTCTGCCGCGTGTAGGCTGGAGCTGCTTC-3′ for *deltaC::mCherry* fusion. BAC transgenesis was performed as described elsewhere^48^,^49^. BAC DNAs were purified with QIAGEN Large-Construct Kit (QIAGEN, Netherlands). *TgBAC(vsx1:GFP)* embryos at the one-cell stage were injected with 100 pg of the purified BAC DNA and 25 pg of *Tol2* transposase mRNA. To detect DeltaC–mCherry fusion protein on V2 cells, two-colour images were acquired sequentially by line using Gallium-Arsenide-Phosphide detectors equipped on Nikon confocal microscope A1R (Nikon, Japan) for observation of DeltaC–mCherry fusion protein localization dynamics during mitotic cell rounding or FVMPE-RS multiphoton laser scanning microscope (Olympus, Japan) for the modification of the shape of V2 cells expressing DeltaC–mCherry fusion protein. For *deltaC*-mutant rescue experiments, the *deltaC::mCherry* BAC DNA construct and *Tol2* transposase mRNA were co-injected into *TgBAC(vsx1:GFP)*^*nns5/−*^; *deltaC*^*tit446/tit446*^ embryos.

### Immunoprecipitation analysis

The injected embryos were solubilized in a Triton X-100 lysis buffer and incubated with anti-mCherry polyclonal antibody (Abcam, UK) for 2 h at 4 °C and immunoprecipitated using protein-G Sepharose beads (GE Healthcare, USA). Proteins were resolved in a 10% acrylamide gel and transferred to a polyvinylidene difluoride membrane for western analysis. Blots were probed with anti-mCherry antibody (polyclonal antibody) or anti-DeltaC antibody (zdc2). Anti-β-tubulin (KMX-1, Merk Millipore, Germany) was used as the input control (Supplementary Fig. 6).

### Luciferase reporter assay

To assess the signal transduction activity of DeltaC–mCherry fusion protein, the following assay was performed. Genomic DNA fragments encoding the wild-type *deltaC* allele and the *deltaC::mCherry* fusion were amplified by PCR from the *deltaC* BAC clone DKEY-46I14 and the *deltaC::mCherry* BAC DNA construct, respectively, and inserted into the pCS2P+ vector. For a negative control experiment, the mCherry sequence was cloned into the pCS2P+. NIH3T3 cells (1 × 10^6^) were transfected with DeltaC, DeltaC::mCherry or mCherry expression vectors (0.5, 1 and 2 μg). Reporter plasmids of Cignal RBP-Jk Reporter (luc) Kit (QIAGEN, Netherlands) were transfected into Lunatic fringe- and Notch1-expressing NIH3T3 cells (LfnNotch1-3T3)^50^ according to the manufacturer's instructions. Twenty-four hours after transfection, LfnNotch1-3T3 cells were split into 2 × 10^4^ cells in 96-well plates. These cells were co-cultured with DeltaC-, DeltaC::mCherry fusion- or mCherry-expressing NIH3T3 cells (2 × 10^4^) for 24 h. The firefly and the *Renilla* luciferase activities were determined using Dual-Reporter Assay System (Promega, USA).

### Transient notch reporter assay

A unit of 25 pg of T2KTp1bglob:hmgb1-mCherry plasmid (TP1-mCherry)^51^ and 100 pg of the *deltaC::mCherry* BAC DNA construct (Dlc::mCherry) were co-injected into *TgBAC(vsx1:GFP)*^*nns5/−*^; *deltaC*^*tit446/tit446*^ embryos along with 25 pg of *Tol2* transposase mRNA. The injected embryos were raised to prim-5 stage (24 h.p.f.). In this triple transgenic line in the DeltaC null mutant background, the nuclear-mCherry level driven by TP1-mCherry and the membrane-mCherry level driven by *deltaC::mCherry* BAC DNA construct (Dlc::mCherry) are expected to represent Notch activity and cell-surface DeltaC levels, respectively, in the GFP-positive (because of *TgBAC(vsx1:GFP)* transgene expression) V2/V2a/V2b cells. Paired V2 cells in the embryos injected only with TP1-mCherry and *Tol2* mRNA were used to quantify the level of nuclear of mCherry (that is, Notch signal activity). Embryos were fixed and processed for whole-mount immunostaining to detect the expression of DeltaC–mCherry fusion protein and mCherry protein reporter. Paired V2 cells positive for both membrane- and nuclear-mCherry expression were subjected to quantitative analyses. Using 3D object counter and 3D Roi manager, the intensity sum of membrane- and nuclear-mCherry was calculated. The nuclear-mCherry intensity was calculated using embryos injected only with TP1-mCherry and *Tol2* mRNA. The membrane-DeltaC level was calculated by subtracting the intensity sums of membrane- and nuclear-mCherry by the intensity sum of the nuclear-mCherry. According to the principle of Notch signalling, mCherry reporter expression should depend on the activity of Delta protein on adjacent sister cell membrane. The intensity sum of the nuclear-mCherry (*y* axis) was plotted against that of the membrane-mCherry (*x* axis) for each sister V2 cell (GFP-positive), demonstrating the lower Notch-signal activity in the DeltaC-deficient V2 cells (□) and the higher Notch-signal activity in the DeltaC-deficient V2 cells where DeltaC::mCherry fusion proteins are expressed (▪) (Fig. 6b, right panel).

### Drug treatment

*TgBAC(vsx1:GFP)* embryos were exposed to drugs at the shield stage (for H89 and U0126) or 15-somite stage (for nocodazole, latrunculin and blebbistatin). The embryos were treated with these drug-containing media until 17-somite stage. The concentrations of the drugs were 100 μM (H89, U0126), 0.5 μg ml^−1^ (nocodazole), 0.4 μM (latrunculin) or 50 μM (blebbistatin).

### Whole-mount single-molecule FISH

Whole-mount single-molecule FISH^52^ was carried out as follows: dechorinated embryos fixed in 4% paraformaldehyde in PBS for 2 h at room temperature were washed with PBS twice and then incubated at −30 °C in methanol for 30 min. The fixed and dehydrated embryos were rehydrated by washing them sequentially with 50% methanol/50% PBST (0.1% Tween-20 in PBS) and then 100% PBST at room temperature for 5 min each. The embryos were then incubated in prehybridization buffer (10% formamide, 2 × SSC, 0.1% Triton X-100, 0.02% BSA and 2 mM Ribonucleoside Vanadyl Complex (New England Biolabs)) at 30 °C for 5 min. The embryos were incubated in the hybridization mix at 30 °C in dark overnight. The hybridization mix was freshly prepared by diluting *deltaC* probe stock solution (25 mM) to 1:100 in hybridization buffer (prehybridization buffer+10% dextran sulfate). After the hybridization, the embryos were washed twice with wash solution (10% formamide, 2 × SSC and 0.1% Triton X-100) at 30 °C for 30 min each. The embryos were mounted on slide glass with ProLong Gold antifade reagent with 4,6-diamidino-2-phenylindole (Life Technologies) for imaging analyses. The *deltaC* probes (Supplementary Table 2) were designed, synthesized and labelled with TAMRA by Biosearch Technologies.

### A computational model with cellular Potts model

In the model, the cell division orientation was assumed to be established by the pulling force generated by the cell-geometry-sensing microtubule-based system anchored to membrane-associated actin fibres (that is, cell cortex; Fig. 3c). To develop a model for the experimentally observed stochastic yet biased asymmetric fate determination, a cell shape-sensing mechanism was assigned to the lateral inhibition signalling system (Fig. 3a). In the lateral inhibition system, Notch activity provides an instructive signal for fate determination. This instructive signal is induced in the cell on the right by the direct engagement of the Notch receptor on the surface with Delta ligand on the directly contacting cell on left (Fig. 3a). This high instructive signal mediated by Notch inhibits the ‘expression' of Delta in the cell on the right reducing the number of Delta on the surface of this cell (Fig. 3a). The reduced number of Delta results in the decreased Notch activity in the cell on the left due to less engagement of Delta to Notch on the left-side cell (Fig. 3a). Then, the reduced Notch activity in the cell on the left is insufficient to suppress the expression of Delta in the cell on the left, thus leading the maintained high level of Delta on the left-side cell (Fig. 3a). Therefore, this lateral inhibition system is extremely effective as only a small quantitative difference in the initial amount of Delta between the two neighbouring cells already suffices for imposing the mutually exclusive and stable binary fates on the adjacent two cells. To this lateral inhibition signalling system, we assigned a new mechanism to account for the experimentally observed shape-directed fate determination (Fig. 3b). Delta is initially localized in a polarized fashion according to the asymmetry of the *in silico* V2 cell shape; The spiky end of (+) side of the long axis has the highest probability of having Delta particles because it is the farthest away from the centre of mass of the cell (***C***_**mass**_), while other area of cell surface has lower probability according to the distance from the ***C***_**mass**_. This results in the polarized distribution of Delta particles according to the direction of the asymmetric elongation of the *in silico* V2 cell shape. As the cell undergoes mitotic rounding, Delta exhibits dynamic movement and spreading on the cell surface (Fig. 3b), resulting in the dilution of its initially polarized localization effect. To simplify the model, we ignore the turnover of the surface Delta particles and the number of Delta particles is fixed during mitotic rounding. When V2 cell divides, the lateral inhibitory interaction sets off. The presence of more Delta particles in a daughter cell induces stronger activation of Notch in the other contacting daughter cell by Delta–Notch trans-engagement at the cell surfaces, thus the former assuming the default V2a fate, and the latter acquiring the induced V2b fate, in accordance with the lateral inhibition system (Fig. 3b). The daughter cell on the (+) side of V2 has higher probability of receiving more Delta, thus preferentially acquires V2a fate, as that is the side where Delta was initially more concentrated due to the V2 cell shape asymmetry (Fig. 3b).

The model was developed using a CPM that was extended with an agent-based model of randomly walking, membrane-bound particles (Supplementary Fig. 3a–e) and an ODE model for the lateral inhibition system. We used the CPM for three reasons: (1) the CPM naturally represents the dynamics of complex cell shape changes in three dimensions; (2) the CPM is readily extended with additional rules to integrate events from multiple scales (for example, cell shape changes, intracellular activities and so on); and (3) the CPM is a stochastic model, thus allowing the simulation of probabilistic events of cell behaviours such as those observed in this study.

In CPM, the space is divided into discrete lattice sites, 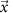, which each contain a discrete number, 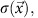 or *CellId* to identify an individual biological cell or extracellular material (for example, medium). One biological cell covers multiple lattice sites, such that a (usually connected) patch of lattice sites, 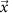, with identical *CellIDs*, 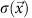 represents a biological cell. The cells' volume *V*(*σ*) is, on average, conserved with small, stochastic deviations due to membrane fluctuations and exchange of materials with the environment. The cells minimize their area of contact with the medium (and with adjacent cells, if present). For this, each interface between two cells or a cell and the medium represented by two adjacent lattice sites with unequal *CellIDs*, that is,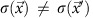, is associated with a positive, interfacial energy, 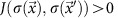. In addition, we use a surface area constraint to represent cortical tension during mitotic rounding. Together these processes define the following Hamiltonian:


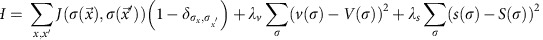


where *v*(*σ*) and *s*(*σ*) are the current cell volume and surface area, respectively, parameters *V*(*σ*) (target volume) and *S*(*σ*) (target surface area) represent resting volume and resting surface area of the cells, respectively, *λ*_*v*_ and *λ*_*s*_ are elasticity parameters, respectively, regulating the volume constraint and cortical tension, and 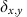 is the Kronecker delta. To mimic random membrane fluctuation, we minimize *H* using a Metropolis algorithm: we iteratively select a random lattice site, 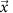, and attempt to copy its *CellID* 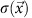 into an adjacent lattice site 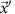 (Supplementary Fig. 3a). We then calculate the change of the Hamiltonian, 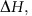 that would occur if we performed the copy, and accept it if Δ*H*<0 or else accept the attempt with Boltzmann probability to mimic active motility of the cells


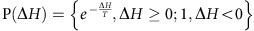


where the cellular temperature *T* represents the degree of active cell motility. In one mcs, the algorithm attempts to perform as many copies as there are sites in the lattice.

The agents represent the membrane-bound particles whose movements are constrained on the membranes of the cells in the CPM. We introduced particles (blue particles that represent cortical force generators and dictate division axis; yellow particles that represent Delta and dictate fate determination) on the cell boundary according to the cell shapes immediately before mitotic rounding (Fig. 3d). We assumed that the membranes near the spiky end of (+) side of long axis have a higher probability of containing particles: to do so the probability that a membrane site contains particle was made according to its distance from the centre of mass of the cell (see below). During mitotic cell rounding, particles perform a random walk on the cell boundary. After the cell rounds up (that is, mitotic rounding), the cell vector (red arrow in Fig. 3d), the sum of unit vectors connecting the centre of mass to blue particles on cell boundary, is calculated to determine the division axis (Fig. 3d, centre panel):


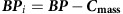



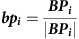



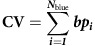


where ***BP*** is a coordinate of a blue particle, *N*_blue_ is the total number of blue particles and **CV** is the cell vector. Cell division takes place along the plane perpendicular to **CV** (Fig. 3d, centre panel).

For the cell-fate determination, the ratio of the number of yellow particle the daughter cell receives initializes the activity of Delta. The initial condition for the activity of Notch is zero.


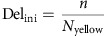



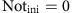


where Del_ini_ and Not_ini_ are the initial condition for the activities of Delta and Notch, respectively, *n* is the number of yellow particle the daughter receives and *N*_yellow_ is the total number of yellow particles. The lateral inhibition and negative feedback loop formed by Delta–Notch interaction is described as ODE as below^21^. To focus on the effect of the initial distribution of the particle and random walk during mitotic rounding, we do not consider other factors of the lateral inhibition except the interaction between Delta and Notch, and decay:


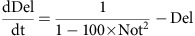



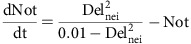


where Del and Not are the activities of Delta and Notch inside a cell, Del_nei_ is the activity of Delta in the neighbouring cell. After cell division, a plugin, BionetSolver^53^, starts to solve the ODEs above. When a daughter cell increases the Notch activity to more than 0.98 and decreases the Delta activity to <0.02, the cell is determined to be V2b (orange in Fig. 3d, right panel) and the other V2a (green in Fig. 3d, right panel). After the cell-fate specification, the ODE model stops. Usually the fate-specification process takes several tens of mcs. With the ODE model for the lateral inhibition system, it is sufficient to incorporate only yellow particles (that is, Delta) as an explicit particle, as the number of yellow particles decides the strength of Notch activity in the daughter cells—the daughter cell receiving higher number of yellow particles show high Delta activity and low Notch activity, and the other daughter cell receiving less yellow particles exhibit lower Delta activity and higher Notch activity—that is, the principle of the lateral inhibition system (Fig. 3b). The total mcs is set to 500.

We built the framework as shown in Supplementary Fig. 3b, where three components were added to the basic CPM model: particle initialization (Supplementary Fig. 3c); particle attachment to cell boundary (Supplementary Fig. 3d); and random walking particle (Supplementary Fig. 3e). The initial cell shapes were defined according to the confocal images of real V2 cells. The cubic lattice sites that compose the virtual 3D cells have sides of length 0.5 μm, which is close to the voxel size (0.42 μm × 0.42 μm × 0.89 μm) of the confocal images. Cell boundary lattice sites are defined as lattice sites adjacent to the surrounding environment (Supplementary Fig. 3c, second left). To distribute the initial particles according to the cell shapes, a boundary lattice site (site 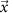 in Supplementary Fig. 3c, second right) is randomly picked up and the distance *d*_1_ from the centre of mass of the cell is calculated. Next, a second lattice site 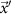 is selected at random from the all lattice sites, and the distance to the origin, *d*_2_, is calculated. If *d*_1_>*d*_2_, a particle is generated at site 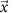 (Supplementary Fig. 3c, second right). Probability of particle generation at site 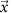 is roughly proportional to *d*_1_^3^ because the probability is given by the volume of eighth part of a sphere of radius *d*_1_ in the lattice space. This can make polarized distribution of particles according to asymmetry of cell shape. Particles are generated until the number designated is reached (Supplementary Fig. 3c, rightmost). After the simulation starts, cell boundary and particles' position are updated after every successful copy attempt (Supplementary Fig. 3d). If a particle is no longer on a boundary lattice site, it will step to one of nearest boundary lattice sites with equal probability. After each mcs, the particles diffuse (Supplementary Fig. 3e). For isotropic diffusion behaviour, a particle moves to one of available lattice sites with equal possibility. The available lattice sites should satisfy two requirements: (1) they should be on current cell boundary; and (2) the distance to them is in the range (*s−*0.5 × *L*, *s+*0.5 × *L*), where *s* is step size of diffusion and *L* is lattice site length.

To recapitulate the mitotic rounding process before the cell division in the model, we set the target volume *V*(*σ*) equal to the initial volume of the cell, and set the target surface area *S*(*σ*)=4*πr*^2^, with *r* the radius of the sphere with volume *V*(*σ*). Under this condition, cells gradually change the initial shape to an approximate sphere shape because the sphere has the smallest surface area among all enclosing objects for a given volume. When current surface area reaches a given value (*S*_division_), a cell divides along the cell vector, which is the sum of directional unit vectors connecting the centre of mass to particles on boundary lattice sites. The detailed parameters are shown in Supplementary Table 1. Particle numbers were set such that they reached a concentration of 5.0 × 10^−3^, 2.5 × 10^−2^, 5.0 × 10^−2^, 0.25 and 0.50 particle per lattice site. After the simulation runs, the cell vectors and the number of particles in the daughter cells were stored in text files and used for analysis. As initial cell shapes, six kinds of shapes (three symmetric and three asymmetric) were tested.

To find parameter values that enable the observed bias in V2 cell asymmetric cell division, we ran the simulations 12 times for each cell shape. As a result, we found that, at a concentration of 5.0 × 10^−2^ particle per lattice site, and with a diffusion rate of 1 lattice site length per mcs (ratio of the concentration to the diffusion rate is 5.0 × 10^−2^), the model best fitted to the experimentally observed bias in division orientation. At a concentration of 5.0 × 10^−2^ particle per lattice site and with a diffusion rate of 2 lattice site length per mcs (ratio of the concentration to the diffusion rate is 2.5 × 10^−2^), the model best fitted to the experimentally observed bias in fate decision. These parameter conditions were found to be within the physiologically relevant ranges. It has been previously estimated that ∼50 cortical force generator exist in *Caenorhabditis elegans* egg^22^. In our computational model, the optimal particle concentration recapitulating the observed bias of the V2 cell division was 2.5–5.0 × 10^−2^ particle per lattice, which corresponds to about 80–160 particles per cell, which is slightly higher than the number of known cortical force generator in *C. elegans* egg but within the approximately same order of magnitude. The optimal diffusion coefficient of the particle in our computational model that mimics the bias of the asymmetric V2 cell division is 1–2 lattice site length per mcs. The average division time of the real cell and computational cell are 21 min and 215 mcs, respectively, thus 1 mcs corresponds to ∼6 s. Since each lattice site length in our CPM is side 0.5 μm long, the diffusion rate of 1–2 lattice site length per mcs corresponds to 1.0–4.2 × 10^−10^ cm^2^ s^−1^ in real situation, estimated by the equation below:


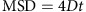


where MSD is the mean squared displacement, *D* is diffusion coefficient and *t* is time. This is within the range of known diffusion coefficient of diffusible membrane proteins, that is 10^−8^ to 10^−11^ cm^2^ s^−1^.

Following the parameter fitting, the two particles (blue and yellow) that each moved independently were integrated into a single *in silico* cell, which was used for the *in silico* experiments. All computational simulations were performed using CompuCell3D (ver. 3.6.2) (http://compucell3D.org)^54^ with custom-made extensions for the random walking boundary particle algorithms written in C++. The scripts can be made available on request.

### Statistical analysis

The *χ*^2^-test is used to determine whether there is a significant difference between the observed frequencies and the expected frequencies. To test the observed frequencies of cell division orientation and fate assignment between daughter cells, we applied the *χ*^2^-test. The chi square statistic is defined as below:


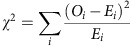


where *χ*^2^ is the chi square statistic, *O*_*i*_ is an observed frequency and *E*_*i*_ is an expected frequency. *χ*^2^ is used to calculate a *P* value by comparing with a chi-squared distribution using Excel. The numbers of degrees of freedom are 2 for cell division orientation and 1 for V2a/b fate specification. In all experiments, *P* values are calculated and shown as *P<0.05, **P<0.01 in the figures.

### Data availability

The data that support the findings of this study and the scripts for all computational simulations are made available from the corresponding author upon request.

## Additional information

**How to cite this article:** Akanuma, T. *et al*. Memory of cell shape biases stochastic fate decision-making despite mitotic rounding. *Nat. Commun.* 7:11963 doi: 10.1038/ncomms11963 (2016).

## Supplementary Material

Supplementary Information Supplementary Figures 1-13, Supplementary Tables 1-2

Supplementary Movie 1 Time-lapse imaging of the neural tube region in Tg(*vsx1:GFP*) embryo. This movie shows V2 progenitor cell development from 3rd to 8th somite level of spinal cord in zebrafish embryo. V2 cells are labelled with GFP driven by *vsx1* promoter. All V2 cells divide once. Dorsal view. Anterior side is on the left. This movie corresponds to Supplementary Fig. 1d. Time interval = 5 min.

Supplementary Movie 2 Time-lapse imaging of a single V2 progenitor cell. This movie shows cell shape change of a single V2 progenitor cell over time. Before cell division, V2 cell rounds up (mitotic rounding). Division orientation is along the long axis of the cell. Dorsal view. Apical side is on the left. This movie corresponds to five panels from left in Fig. 1a. Time interval = 5 min.

Supplementary Movie 3 Simulation of V2 cell fate decision-making with asymmetric shape. Following the rounding and division of the in silico V2 cell, a daughter cell receiving more yellow particles assumes the V2a fate and remains in green color, while the other with less yellow particles acquires the V2b fate and turns to orange color. Particle concentration is 5.0 × 10-2 particle/lattice site. Diffusion rate is 1 lattice site/mcs for blue particles and 2 lattice site length/mcs for yellow particles. This movie corresponds to the one with A_long_=0.063 in Fig. 3e. The movie includes blue particles orienting the division (not included in the Fig. 3e) and the 3D rotation.

Supplementary Movie 4 Simulation of V2 cell fate decision-making with relatively symmetric shape. Fate-determination of V2 cell with relatively symmetric shape under the same condition as the Supplementary Movie 3 is shown. In this movie, the (+)-side daughter cell chooses the V2b fate, whereas the (-)-side daughter cell chooses the V2a fate. This movie corresponds to the one with A_long_=0.002 in Fig. 3e. The movie includes blue particles orienting the division (not included in the Fig. 3e) and the 3D rotation.

Supplementary Movie 5 Dynamics of DeltaC::mCherry fusion protein localization during mitotic rounding. The real-time imaging shows that DeltaC::mCherry fusion protein spreads over V2 cell surface during mitotic rounding. Time interval is 1 minute. This movie corresponds to the upper panels in Fig. 5c.

Supplementary Movie 6 Cell shape change induced by femtosecond laser causes the translocation of DeltaC::mCherry fusion protein. The whiteout of the movie image is the time when the V2 cell was laser-irradiated. DeltaC::mCherry fusion protein translocates and is enriched on the (+)-side of the newly formed long axis (on the right in the movie). The old (+)-side is on the left side of the image. After the laser irradiation, the real-time imaging was recorded with the time interval of 1 minute. This movie corresponds to the left panels in Fig. 5d.
