# Supplementary material for: Memory of cell shape biases stochastic fate decision-making despite mitotic rounding
Source: Nat Commun. 2016 Jun 28;7:11963. doi: 10.1038/ncomms11963 (PMC4931277; doi:10.1038/ncomms11963)
Supplement: Supplementary Information — Supplementary Figures 1-13, Supplementary Tables 1-2 [file ncomms11963-s1.pdf]

Supplementary Figures

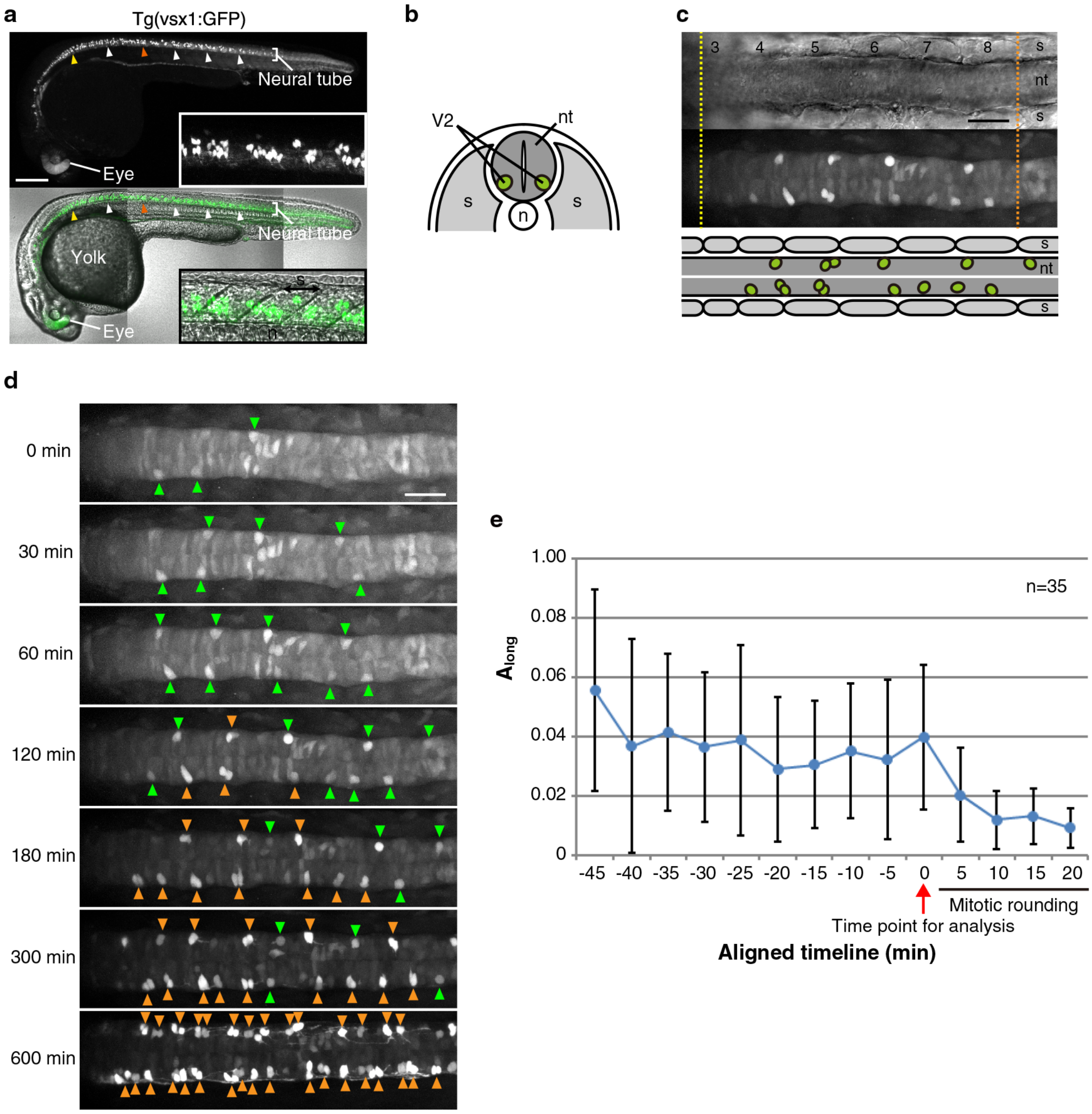

Supplementary Figure 1. V2 cells in neural tube.

(a) Tg(*vsx1*:GFP) zebrafish embryo at the prim-5 stage (24 hpf). The fluorescein image (upper panel) and the one merged with the bright field image (lower panel) are shown (lateral view with anterior side on the left). In the boxes shown is the higher magnification of the trunk neural tube region where GFP is expressed in V2 cells (arrowheads). A yellow arrowhead indicates the anterior end of the third-somite level and an orange arrowhead indicates the

posterior end of the eighth-somite level. A double arrow in the boxed bright field image indicates the length of a single somite. n: notochord, s: somite. Scale bar, 200 μm. (b) Schematic diagram of the cross section of the trunk region of zebrafish embryo. V2 cells (green) are located at the edge of ventral neural tube. (c) Dorsal view of the neural tube region of Tg(*vsx1*:GFP) embryo. Bright field image (upper), fluorescein image (middle) and schematic diagram (lower) are shown. Anterior is on the left. V2 cells are shown in green in the schematic diagram. Yellow and orange dotted lines indicate the anterior end of the third somite and the posterior end of the eighth somite, respectively. nt: neural tube, n: notochord, s: somite. Scale bar, 50μm. (d) Time-lapse images of GFP^+ V2 cells from the 14-somite stage (0 min) to the prim-5 stage (600 min). See also Supplementary Movie S1. Dorsal view. Anterior side is on the left. V2 cells prior to the cell division (green arrowheads) and paired V2 daughter cells (orange arrowheads) are indicated. Scale bar, 50μm. (e) Changes in the degree (A_{long}) of asymmetry of V2 cells (n=35) over time are shown. The time when the cells enter into mitotic rounding phase is set at zero. Time points for each cell are adjusted so that the time-point when the cell shape was determined immediately prior to mitotic rounding phase is set at 0 (zero), the time course for all cells are aligned to this 0 time-point and indicated as "aligned timeline" in the graph. Error bars and standard deviations are indicated at each time point.

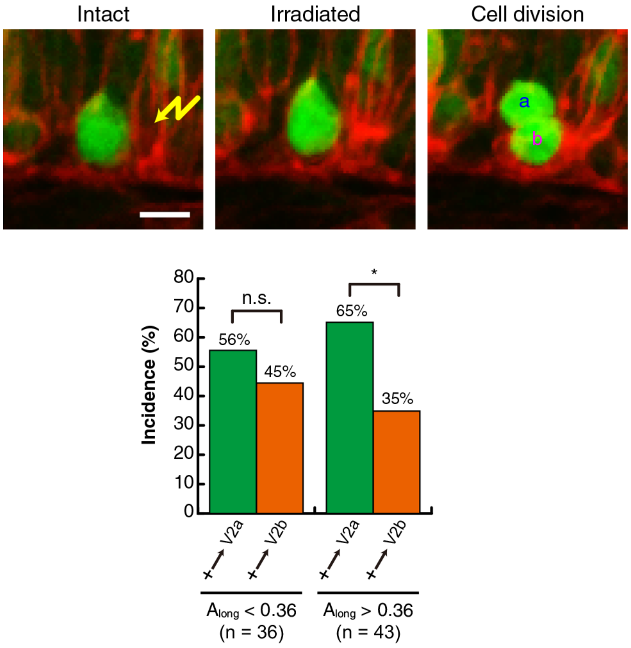

Supplementary Figure 2. No influences of neighboring non-V2 cells on V2 cell fates.

Femtosecond laser irradiation on neighboring non-V2 cells. Neighboring cells (labeled by membrane-bound mCherry: mCherry::CAAX) are irradiated (yellow arrow in the left panel), damaged (center panel), the daughter cell fates are determined (right panel and the lower panel graph). The bottom graphs are the quantifications of the results. n.s.: not significant, $*p<0.05$, $**p<0.01$ (chi-squared test). Scale bar, 10μm.

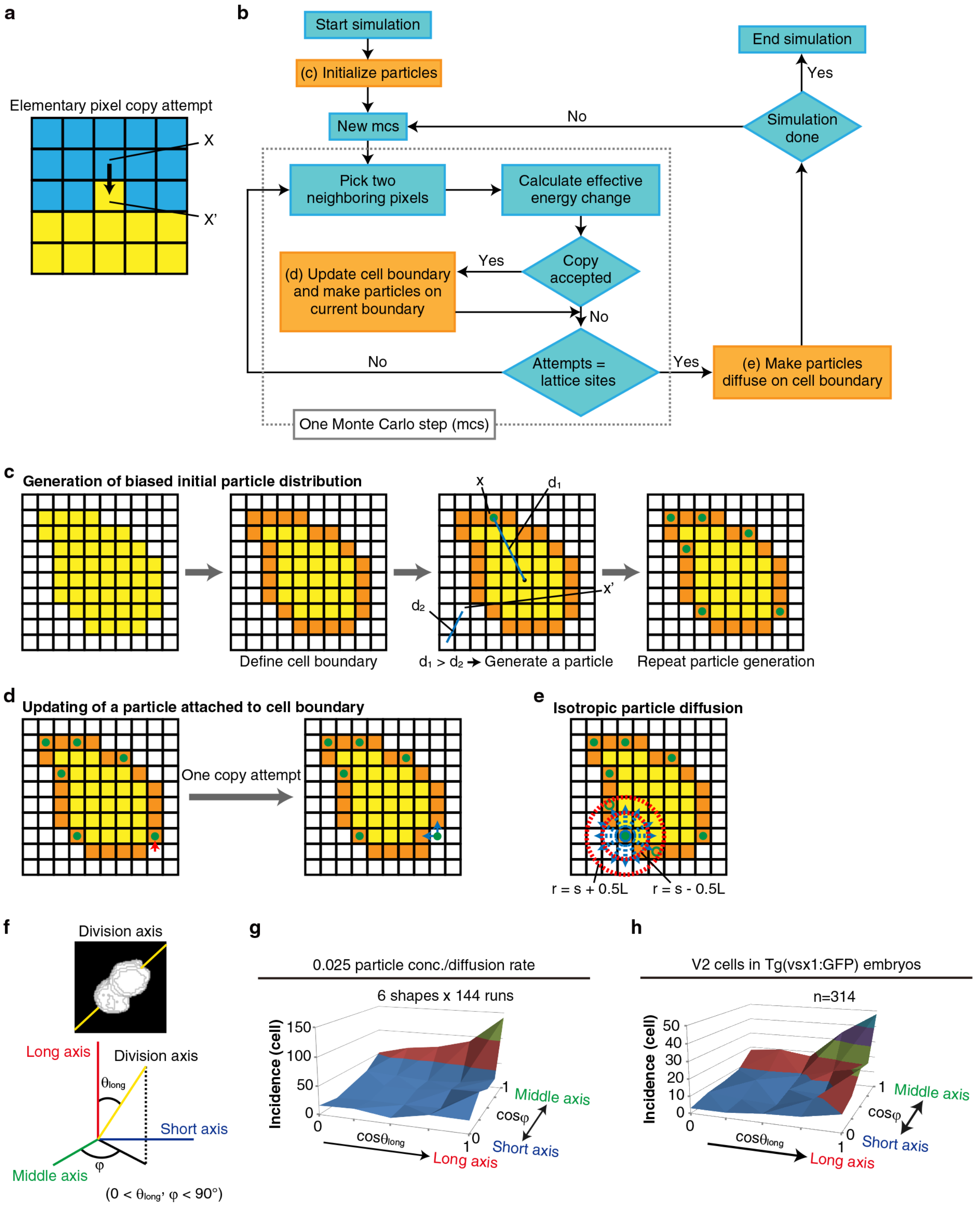

Supplementary Figure 3. Basic and extended Cellular Potts Model.

(a) Diagram representing a copy attempt in the cellular Potts model (CPM). To facilitate the visualization, lattice space is represented in 2D square grid. The "blue" lattice site located in site, $\vec{X}$ is going to replace "yellow" lattice site in site, $\overrightarrow{X'}$ (arrow). (b) The flowchart of the randomly walking diffusion particle model in CPM. The blue components are organized to be

a typical process of CPM, while the orange components are new components designed for our randomly walking boundary particle model. (c) Diagram representing particle initialization. A cell consists of connected patches of yellow lattice sites (leftmost). The cell boundary (orange lattice sites) is defined with respect to the nearest neighbor lattice sites to surrounding white (medium) lattice sites (second left). To generate a particle, a lattice site, $\vec{x}$, is selected at random from the cell boundary. If the distance from the center of mass, d_1, is larger than the distance, d_2, between the origin and a second lattice site, $\overrightarrow{x'}$, randomly selected from all lattice sites, a particle is generated on the cell boundary lattice site $\vec{x}$ (second right). In this way particles are most likely generated at locations closest to the spikes, which are farthest from the center of mass of the cell. The process is repeated until the number designated is reached (rightmost). (d) Diagram representing the updating of cell boundary and a particle after one copy attempt. When a boundary lattice site with a particle is copied by the medium lattice site (left panel, red arrow), the left neighboring lattice site is updated to be a new boundary lattice site (right panel). The particle in old boundary lattice site will move to the up or left neighboring lattice site with equivalent probability (blue arrows). (e) Diagram representing particle diffusing on cell boundary which consists of a number of lattice sites. The diffusion step size (s) is 2 times of lattice site length (L) while we search for the available lattice sites between 1.5 and 2.5 times of lattice site length (red dot circles), enabling isotropic diffusion behavior (blue dot arrows). In this example, two lattice sites (green circle) are available for a particle (green) to diffuse. r: radius. (f) Representation of division axis orientation relative to three principle axes. (g) The probability distribution of the division axis of the in silico cells of six different A_{long} values (0.002, 0.024, 0.031, 0.052, 0.063, 0.092) shown as 3D surface plot. (h) Probability distribution of division orientation of real V2 cells shown as 3D surface plot.

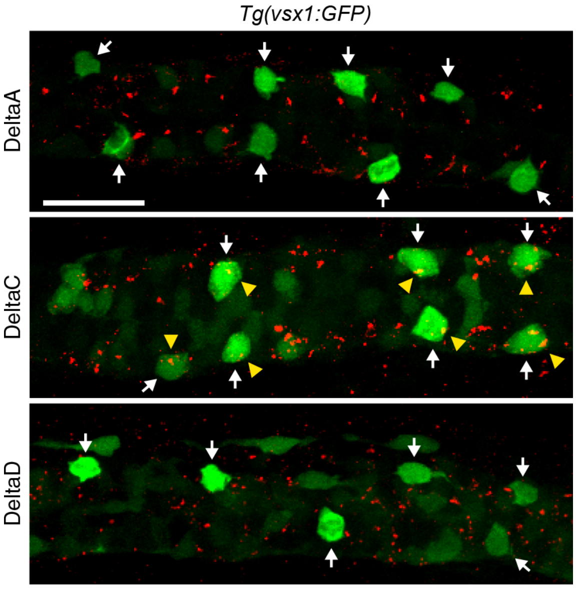

Supplementary Figure 4. DeltaC, but not DeltaA or DeltaD, is expressed in V2 cells.

Embryos (16- to 18-somite-stage) stained with antibodies against DeltaA, DeltaC and DeltaD. The expressions of Delta proteins are detected as dot-like signals (red). V2 cells (green) are indicated by arrows. The DeltaC protein expression in V2 cells is indicated by yellow arrowheads. The expressions of Delta proteins are also detected in non-V2 cells (dot-like red signals outside green V2 cells). Cells of faintly green are non-V2 cells where there is some weak leaky expression of GFP. We could not evaluate DeltaB as no reliable antibody reagent for DeltaB immunostaining was available. Scale bar, 30 μm.

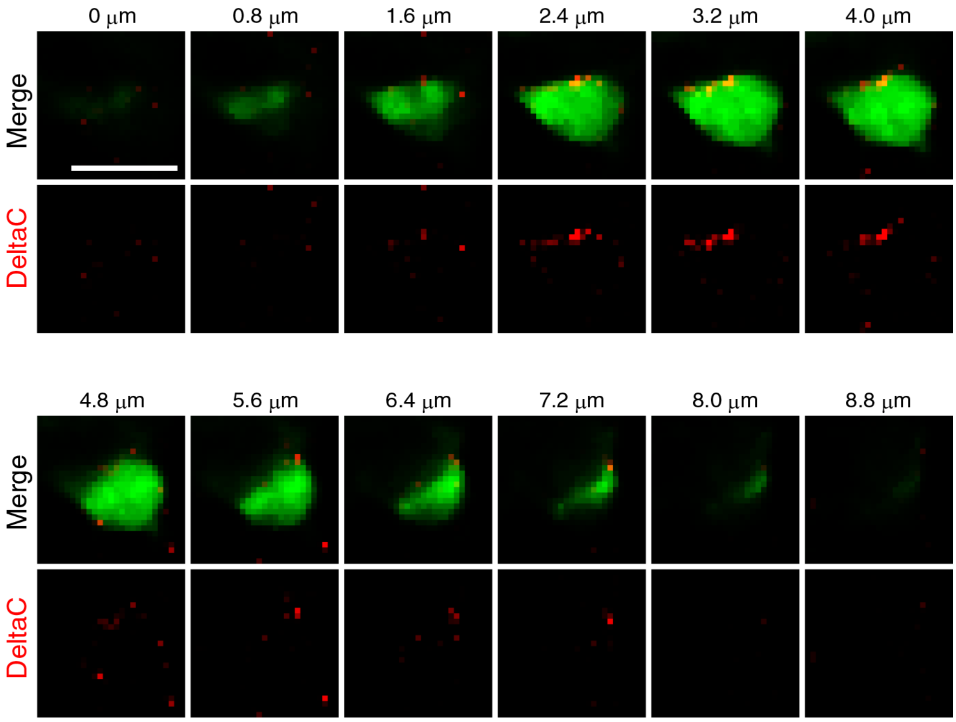

Supplementary Figure 5. Optical serial sections of V2 cell stained for DeltaC proteins.

Zebrafish embryo stained with anti-DeltaC antibody was mounted for confocal imaging and optical serial sections of every 0.8μm are taken. Cytoplasm is highlighted by GFP (green). Cell surface DeltaC stainings are shown in red. Scale bar, 10μm.

a

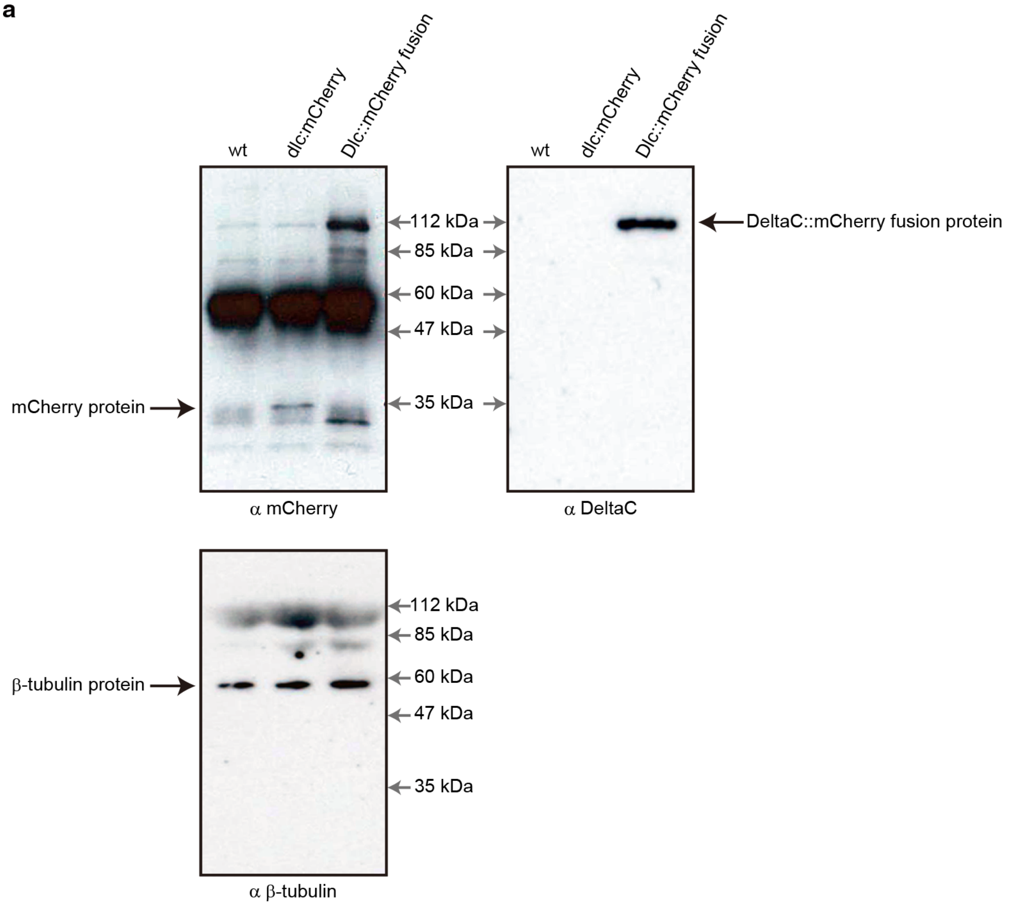
wt
dlc:mCherry
Dlc::mCherry fusion
112 kDa
85 kDa
60 kDa
47 kDa
35 kDa
DeltaC::mCherry fusion protein
mCherry protein
α mCherry
α DeltaC
β-tubulin protein
α β-tubulin

b

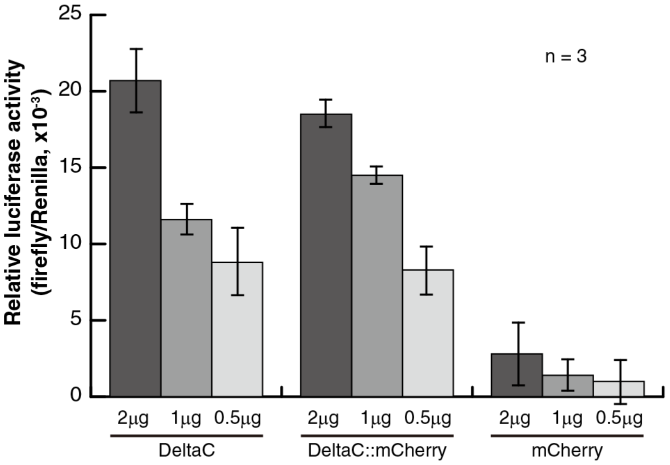
Relative luciferase activity (firefly/Renilla, x10^{-3})
25
20
15
10
5
0
n = 3
2μg 1μg 0.5μg
DeltaC
DeltaC::mCherry
mCherry

c

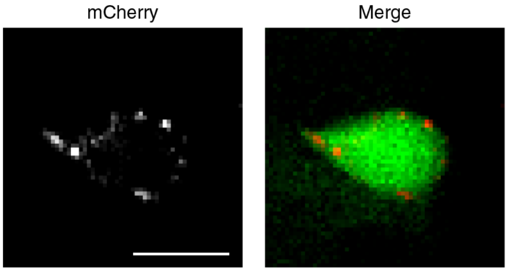
mCherry
Merge

Supplementary Figure 6. Characterization of DeltaC::mCherry fusion protein.

(a) Western blot analysis of DeltaC::mCherry fusion protein immunoprecipitated with anti-mCherry antibodies from uninjected wild type (lane 1 in each panel), dlcC:mCherry control BAC-injected (lane 2 in each panel) and DeltaC::mCherry-overexpressed embryos (lane 3 in each panel). The band of the expected size for mCherry is detected in the lane 2 in the left gel panel (anti-mCherry). The band for DeltaC::mCherry fusion protein is detected in the lane 3 in both left (anti-mCherry) and right (anti-DeltaC) gel panels. The size markers are shown between the two panels. (b) Activation of Notch-signaling by DeltaC::mCherry fusion protein expression. Error bars indicate standard deviations. n=3. (c) Cell surface localization of DeltaC::mCherry fusion protein. Scale bar, 10μm.

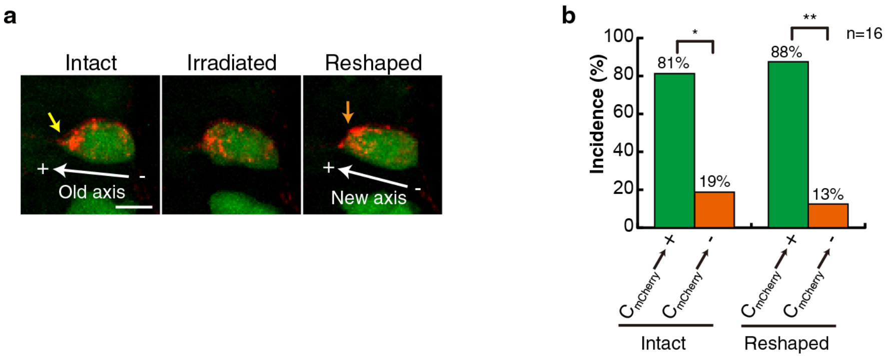

Supplementary Figure 7. No influence of femtosecond laser irradiation on DeltaC::mCherry Fusion Protein localization.

(a) A case where the laser-irradiated V2 cell maintains the same orientation of the long axis as the one prior to the irradiation (difference in angle between the old axis and new axis is less than 45°). A V2 cell is irradiated with femtosecond laser at the tip of the long axis (yellow arrow). Scale bar, 10μm. (b) The irradiation did not alter the orientation of the long axis of the V2 cell, thus both old and new axes are pointing towards the same direction. DeltaC::mCherry fusion protein remains highly concentrated at the tip of the new axis (orange arrow) that is pointing towards the same direction as the old axis prior to the irradiation. *p<0.05, **p<0.01 (chi-squared test).

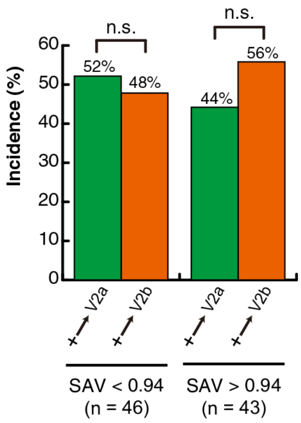

Supplementary Figure 8. No correlation of the biased fates to surface-area-to-volume (SAV) ratio.

The fates for the V2 cells (n=89) with small (<0.94) (left) and large (>0.94) (right) SAV are shown. The average SAV is 0.94 μm^{-1}. n.s.: not significant (chi-squared test).

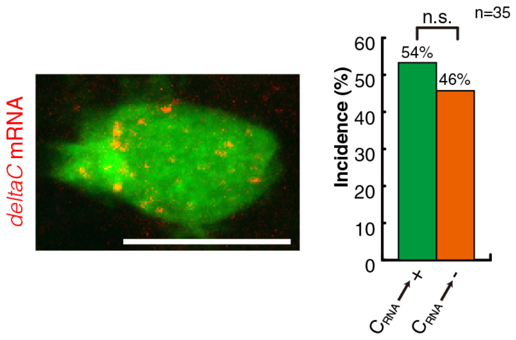

Supplementary Figure 9. Distribution of *deltaC* transcripts in V2 cells.

Whole-mount smFISH for *deltaC* transcripts (red in the left panel) in V2 cells of Tg(*vsx1*:GFP) embryo and the quantification of a total of 35 V2 cells (right graph). n.s.: not significant (chi-squared test). Scale bar, 10μm.

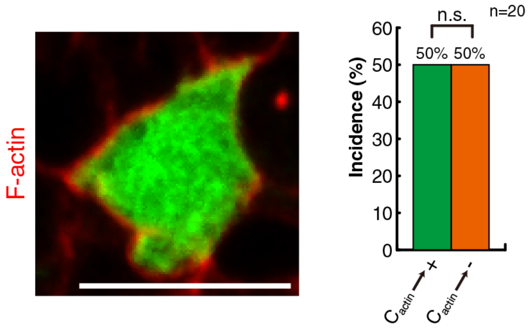

Supplementary Figure 10. Localization of actin fibers in V2 cells.

Typical actin fibers visualized by phalloidin staining (red in the left panel) and the quantification of a total of 20 V2 cells (right graph). n.s.: not significant (chi-squared test). Scale bar, 10μm.

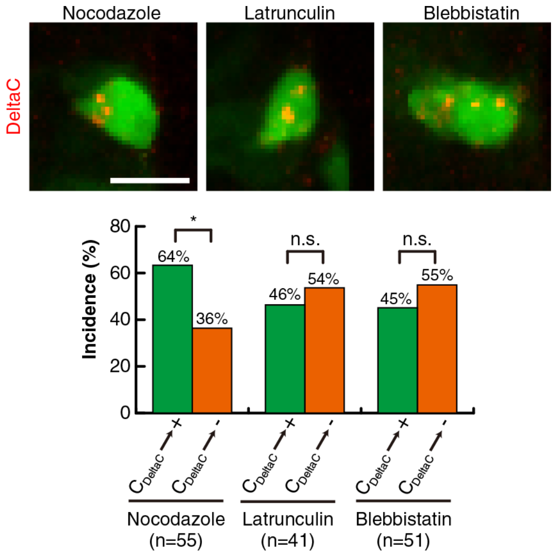

Supplementary Figure 11. Effects of cytoskeleton inhibitors on DeltaC protein localization.

Typical localization of DeltaC proteins (orange dots) in the V2 cells (green) treated with nocodazole (inhibitor of microtubule polymerization), latrunculin (inhibitor of actin polymerization) and blebbistatin (inhibitor of ATPase activity of non-muscle myosin II) are shown (upper panels). Quantification of DeltaC protein localizations in V2 cells treated with each inhibitor is shown as bar graphs (bottom panels). n.s.: not significant, *$p<0.05$ (chi-squared test). Scale bar, 10μm.

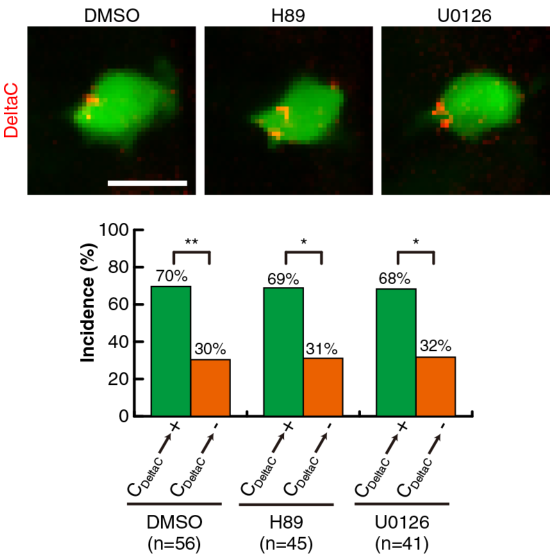

Supplementary Figure 12. Effects of PKA and MEK1/2 inhibitors on Delta protein localization.

Typical localization patterns of DeltaC proteins (orange) in V2 cells (green) treated with PKA (H89) and MEK1/2 (U0126) inhibitors are shown (upper panels). Quantifications are shown as bar graphs at the bottom. DMSO treatment is used as a negative control. *$p<0.05$, **$p<0.01$ (chi-squared test). Scale bar, 10μm.

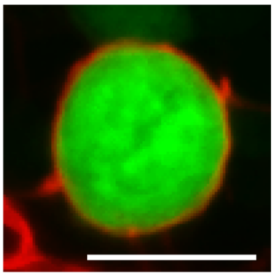

Supplementary Figure 13. Morphology of V2 cells in mitotic rounding phase.

The V2 cell plasma membrane is visualized by membrane-bound mCherry (red). Scale bar, 10μm.

Supplementary Tables

Supplementary Table 1. Model parameters.

Parameter	Definition	Setting
	lattice dimensions	60: 60: 60
L	lattice length	0.5 μm
T	temperature	10
$J_{cell,\ medium}$	bond energy (cell-medium)	2
$J_{cell,\ cell}$	bond energy (cell-cell)	4
V_σ	target volume	initial volume (= $4\pi r^3/3$)
λ_v	volume constraint	0.6
S_σ	target surface area	$4\pi r^2$
λ_s	surface constraint	0.006
$S_{division}$	division condition	$1.45 \times S_\sigma$

Supplementary Table 2. Nucleotide sequences (5' -> 3') of *deltaC* probes for single molecule FISH.

Probe #	Sequence	Probe #	Sequence	Probe #	Sequence
1	atctcaatgtcgcatgtgtt	17	attggtttcgatctcacagt	33	gtggaaccagcaatgttgat
2	actgtgagagtagtccgata	18	gtcgattttcttctcacagt	34	cacgttgttctatgtgttcg
3	gtctgctactgaacgatagc	19	tttgttgcccaaatcgagac	35	gaattctgctatacctcagt
4	tgctcgtgcttgtgaaagac	20	agcagtcgtcaatgtttgtt	36	cttttgtccttagagactgt
5	ggtaatatgagtgcttgcga	21	agaaaccaagcgtgcatgtg	37	aatccttgattcttcagctc
6	cagtatttctgacattccgg	22	gaagtgagtgtagcaggttc	38	gatgaacttcaggatgccta
7	atatacgcactgctggagat	23	agactattaaggctgctgga	39	ccataaggctgattgtttgg
8	atcaaagagacgattcccg	24	ggttagagtaatgaggccta	40	gcaaatactccacagtcaca
9	tattctctgttgactggtca	25	acaatggcagcacagatcac	41	attgcttctcatcacagttc
10	gatagcgagtcttcttttgg	26	acagaatccaggttgtttcg	42	tcacagccagaacaacttgt
11	gaaaagcgcagttggctttg	27	tacctaaagtggaggttggg	43	tcactgccgcataaaactgt
12	cgtggtagaattcatcgcac	28	taaatgggccaccaggaata	44	tgtcactgatagtcagatcc
13	tccaggaaggcaaattctgt	29	cgccatatctttattggaca	45	tgtaactgagctgtagcaca
14	tacagccagacaagcagatg	30	gtcactggaatgtgtgtcta	46	aaggctcttttcagtgtttc
15	gcaagtgcactgaaaaggct	31	ccatcttctgcttatagttt	47	acacaacagcatccatcatc
16	gggcttgtgattagtgcaaa	32	tgtttgtgtgcttttcatca	48	ccagcagtgaatgttgacag
